# Sand fly saliva reprograms skin fibroblasts to enhance arbovirus infection

**DOI:** 10.1016/j.isci.2025.113854

**Published:** 2025-10-25

**Authors:** Yonca Keskek Turk, Ailish McCafferty-Brown, Liam Barningham, Magdalena Jancarova, Petr Volf, Matthew E. Rogers, Akira J.T. Alexander, Çağdaş Kaya, Sandy MacDonald, Maria Grazia Cusi, Alain Kohl, Kave Shams, Clive S. McKimmie

**Affiliations:** 1 School of Medicine, University of Leeds, Leeds, UK; 2 Virus Host Interaction Team, Skin Research Centre, York Biomedical Research Institute, Hull York Medical School, University of York, York, UK; 3 Department of Parasitology, Faculty of Science, Charles University, 128 00 Prague, Czech Republic; 4 Department of Disease Control, Faculty of Infectious and Tropical Diseases, London School of Hygiene and Tropical Medicine, London, UK; 5 MRC University of Glasgow Centre for Virus Research, 464 Bearsden Road, Glasgow, UK; 6 Maltepe University, Faculty of Medicine, Istanbul, Turkiye; 7 Genomics and Bioinformatics Laboratory, Department of Biology, University of York, Heslington, York, UK; 8 Virology Unit, Department of Medical Biotechnologies, University of Siena, 53100 Siena, Italy; 9 Centre for Neglected Tropical Diseases, Departments of Tropical Disease Biology and Vector Biology, Liverpool School of Tropical Medicine, Pembroke Place, Liverpool, UK; 10 Inflammatory Skin Disease Group, Leeds Institute of Rheumatic and Musculoskeletal Medicine, School of Medicine, Faculty of Medicine and Health, University of Leeds, Leeds, UK

**Keywords:** health sciences, medical microbiology, viral microbiology, oral microbiology

## Abstract

Arbovirus transmission by sand flies is a growing public health concern, yet the early skin events shaping infection outcomes remain undefined. We establish a mouse model of Toscana virus (TOSV) infection that incorporates sand fly salivary factors to mimic natural transmission. Saliva from two distinct sand fly genera significantly enhanced infection and promoted neurological signs and joint inflammation, recapitulating key features of human TOSV disease. In the skin, dermal macrophages and fibroblasts were the main infected cell types, but only fibroblasts generated infectious virus. Saliva reprogrammed fibroblasts into a wound-healing state permissive to viral replication, driving local viral amplification, systemic spread, and thereby clinical disease. These findings identify skin fibroblasts as central determinants of host susceptibility and reveal that sand fly saliva actively remodels the skin to exacerbate viral pathogenesis. This work redefines the skin’s role in sand fly-transmitted infection and highlights new targets for therapeutic and vaccine development.

## Highlights

• Sand fly saliva enhances sand fly-borne TOSV and unrelated SFV infection in a mouse model
• Fibroblasts, not macrophages, produce infectious virus in the skin
• Saliva reprograms fibroblasts into a pro-viral, wound-healing state
• Saliva enhanced infection leads to joint inflammation and neurological signs

## Graphical abstract

**Figure:**
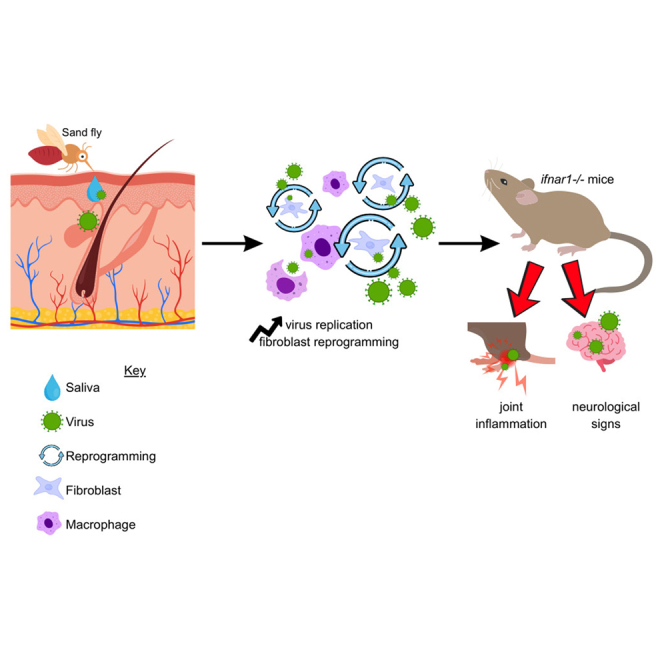

## Teaser

Health sciences; Medical Microbiology; Viral microbiology; Microbiology; Virus: Skin; Vector; Fibroblast; Inflammation; Pathogenesis

## Introduction

Arthropod-borne virus (arbovirus) infections constitute a growing threat to human health as the climate crisis worsens and globalization facilitates their spread to new geographic locations. This includes infections spread by phlebotomine sand flies that are increasingly common in many temperate regions and can act as efficient vectors of various diseases including those caused by both parasites and viruses.^1^^,^^2^^,^^3^^,^^4^ One of the most medically important viruses spread by sand flies is the Toscana virus (*Phlebovirus toscanaense*, TOSV).^5^^,^^6^ TOSV is one of several viruses transmitted by sand flies, each of which have potential to cause widespread outbreaks of disease, such as Sandfly fever Naples virus. However, as the only sand fly-borne phlebovirus known to cause neurological infections in humans, TOSV is now the most significant cause of aseptic acute meningitis and encephalitis, particularly during the warm season in many endemic regions of the Mediterranean Basin.^7^^,^^8^^,^^9^ As the climate warms, cases of infection have now been detected outside this range, as highlighted by the recent detection of autochthonous TOSV meningoencephalitis.^10^ The Chandipura virus is another sand fly-borne neurotropic virus of increasing concern in India.^11^ There are no vaccines or antivirals available for treating or preventing sand fly-borne virus infections. As such there remains a key unmet need to better understand their pathogenesis, transmission dynamics, and host-pathogen interactions.

Arboviruses, including TOSV, are almost exclusively transmitted through the bites of hematophagous arthropods such as mosquitoes and sand flies. These vectors deposit not only virus, but also a complex mixture of salivary components, which for mosquito-borne virus, significantly influence host responses and increases susceptibility to infection for a range of genetically distinct viruses.^12^^,^^13^ This includes salivary factors from *Aedes* and *Culex* mosquitoes that enhance the infectivity of various arboviruses, including *Bunyaviricetes* such as Rift Valley fever virus, Cache Valley virus, and Bunyamwera virus^14^^,^^15^^,^^16^; orthoflaviviruses such as dengue (DENV), West Nile,^17^ and Zika viruses (ZIKV); and alphaviruses such as Semliki Forest virus (SFV)^18^ and chikungunya virus.^19^ Sand flies, as vectors of various pathogens, are perhaps best known as efficient vectors of *Leishmania* parasites. Here too, vertebrate inflammatory responses to sand fly bites, which includes a rapid infiltration of inflammatory myeloid cells, enhances parasite establishment.^20^^,^^21^^,^^22^ However, it is not known what impact sand fly saliva has on viral transmission, or whether host response to sand fly factors could alter susceptibility. In this report, we establish a new mouse model that mimics natural infection by TOSV at the skin inoculation site, which crucially includes co-inoculation with sand fly saliva. We show that saliva from two distinct genera of sand flies transforms vertebrate susceptibility to genetically distinct virus, resulting in enhanced virus replication, dissemination, and the establishment of clinical disease. Here, sand fly saliva reprograms fibroblasts into a wound healing phenotype that inadvertently replicate virus more efficiently. These findings establish a key role of sand fly derived factors in influencing susceptibility to virus, reveal mechanistic insights into this key stage of infection, and identify novel targets for future therapies.

## Results

### Development and use of a TOSV mouse model

TOSV is a human pathogen that does not efficiently infect other mammalian species, including mice. We therefore sought to establish a mouse model that exhibited susceptibility to TOSV infection using a previously characterized strain (1812V) that can efficiently replicate in mouse brain.^23^ We found that following skin inoculation, virus was not able to replicate or disseminate systemically in C57Bl/6 mice, as shown by low quantitates of virus RNA that decreased rapidly post infection, with no evidence of infectious virus by plaque assay, irrespective of cell line used to generate virus (Figures S1B and S1C). Next, we assessed whether TOSV could replicate within mice with suppressed type I interferon (IFN) signaling. Mice given antibodies to block IFNAR1 function (e.g., previously used to establish IFN-sensitive ZIKV infection in mice)^24^ supported limited TOSV infection in a dose-dependent manner (Figures S1D and S1E). In comparison, when *ifnar*-1-null mice that are fully deficient in IFN signaling were infected, they exhibited more robust replication and dissemination of virus, e.g., spleen by 72 h post infection (hpi). In all cases lymph nodes (LNs) appeared refractory to infection (Figure S1F). In summary, infection of *ifnar1*-null mice was used for these studies.

### Sand fly encoded salivary extracts enhance infection with genetically distinct arboviruses in an IFN-independent manner

**Figure 1:**
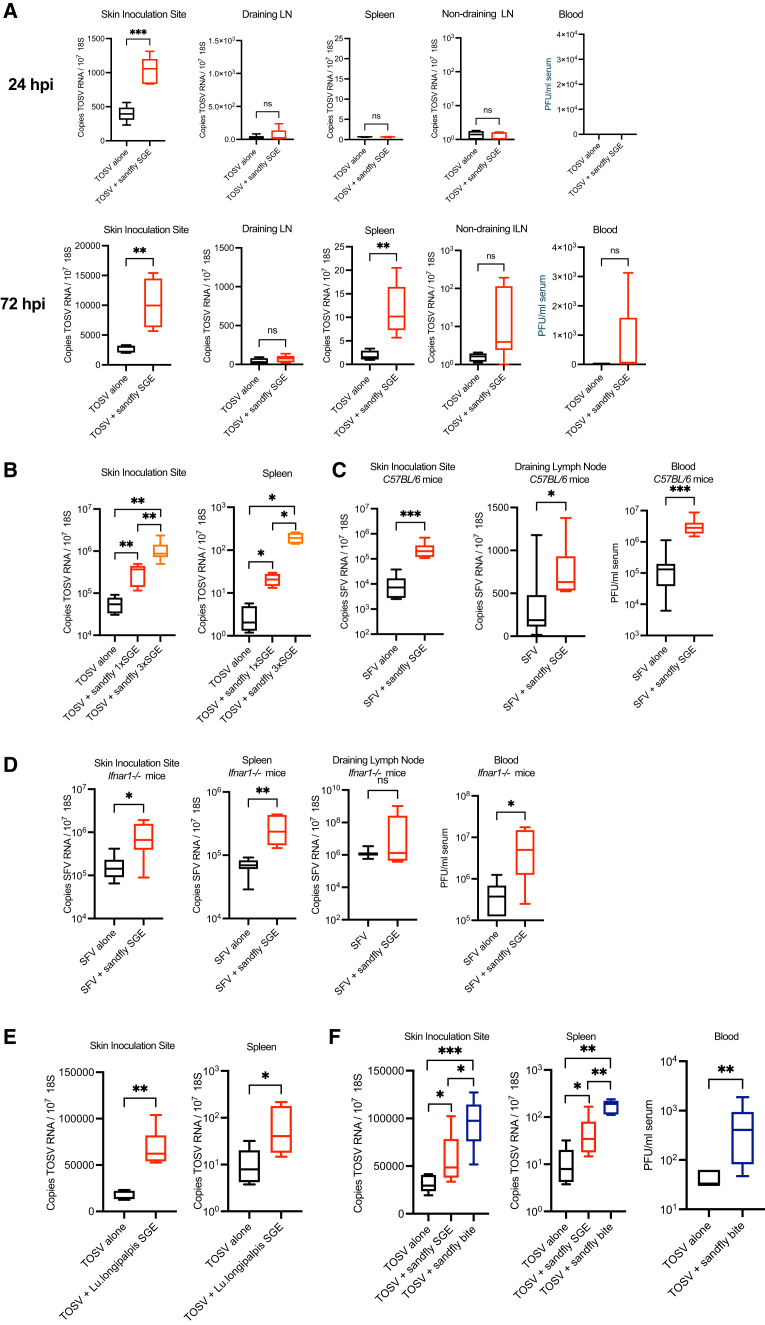
Sand fly SGE and biting enhance susceptibility to TOSV Infection in mice Mouse skin was inoculated with virus, with or without additional saliva gland extract (SGE) in a 1 μL volume by custom-made needle to mimic natural infection by sand fly. (A and B) *Ifnar1*^−/−^ mice (*n* = 5) were infected with 10^5^ PFU TOSV with or without *P*. *perniciosus* sand fly salivary gland extract (SGE). (A) 0 or 1 or (B) 0 to 3 salivary gland extracts per TOSV injection. Tissues were taken at either 24hpi (A) or 72hpi (A and B). (C) C57BL/6 mice (*n* = 8) or (D) *Ifnar1*^−/−^ mice (*n* = 7), were infected with 10^4^ PFU of SFV4 with or without 1 *P*. *perniciosus* SGE and samples obtained at 24 hpi. (E and F) *Ifnar1*^−/−^ mice were infected with 10^5^ PFU TOSV alone, mixed with 1 *Lu. Longipalpis* sand fly SGE, or injected into *Lu*. *Longipalpis* bitten skin (F). All injections and biting were done on the upper side of the left foot (not foot pad). Virus RNA was quantified by qPCR and infectious units in serum defined by plaque assay. Plots show the median value ± interquartile range. ns = not significant, significant *∗p* < 0.05, ∗∗*p* < 0.01, ∗∗∗*p* < 0.001. Ordinary one-way ANOVA were performed for comparisons between more than two groups of normally distributed data, whereas unpaired, two-tail Student’s *t* test were performed for comparisons between two groups.

To determine whether factors from saliva of the primary vector of TOSV in western Europe, *Phlebotomus perniciosus* sand flies, could influence susceptibility to TOSV, we infected mice with either virus alone, or virus mixed with sand fly saliva gland extract (SGE). SGE from *P*. *perniciosus* was obtained by gentle disruption and centrifugation of dissected salivary glands in saline solution, to obtain soluble salivary factors without the inclusion of intracellular contents. Inclusion of SGE derived from just one fly with virus inoculum resulted in enhanced infection of mice, with significantly higher quantities of virus RNA in skin at 24 hpi, which was further elevated by 72 hpi (Figure 1A). Dissemination of virus to spleen and non-draining inguinal LNs was evident by 72 hpi, which was enhanced in mice also receiving SGE. Like most arboviruses, dissemination to spleen is via the blood. However, most mice did not exhibit detectable quantities of infectious virus in blood, although some mice receiving SGE did become viremic, suggesting virus in blood is rapidly cleared. Enhancement of infection by SGE was dose-dependent, with mice receiving the SGE from three sand flies exhibiting the highest quantities of virus RNA (Figure 1B).

To determine whether SGE’s effect on host susceptibility was specific to sand fly-borne TOSV, we next defined whether SGE could also enhance infection with a genetically unrelated arbovirus, SFV. This virus is a mosquito-borne alphavirus within *Togaviridae*, which has the advantage of replicating in both wild type immunocompetent and *ifnar1*-null mice. Here, inclusion of SGE with SFV inoculum resulted in significantly increased quantities of virus RNA in skin and LN, and infectious virus in serum by 10 to 100 folds (Figure 1C). The ability of SGE to enhance virus infection in both immunocompetent and *ifnar1*-deficient mice demonstrated that the mechanism was independent of type I IFN responses (Figure 1D). To define how widely applicable these findings were, we also assessed whether SGE from a genetically unrelated genus of sand fly, *Lutzomyia longipalpis*, could enhance susceptibility to TOSV. Here, *Lu*. *Longipalpis* SGE inclusion with TOSV also resulted in significantly higher quantities of virus RNA in skin and spleen (Figure 1E). Interestingly, injection of TOSV into skin bitten by this genus of sand fly resulted in quantities of virus RNA that were even higher, and notably also enabled efficient establishment of viremia (Figure 1F).

#### Enhancement of virus infection by SGE is unaffected by protein denaturation and microbiota depletion, but not action by RNases

**Figure 2:**
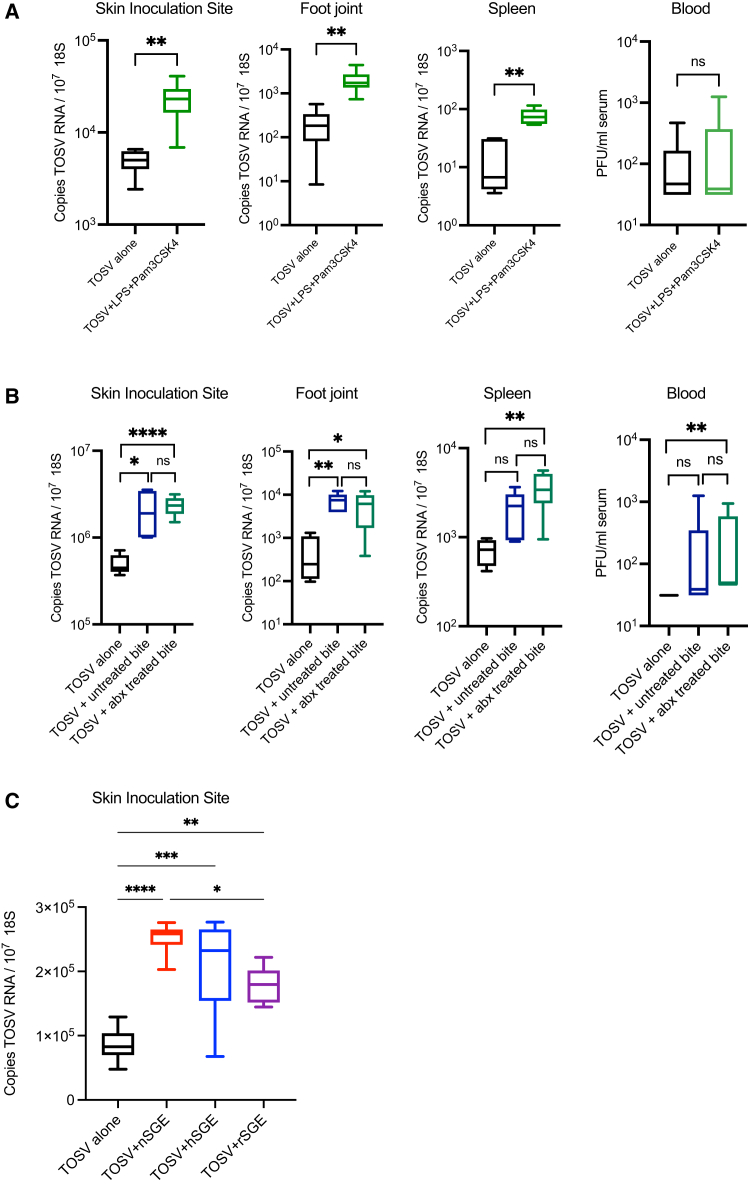
Enhancement of virus infection by SGE is unaffected by protein denaturation and microbiota depletion, but not action by RNases (A) *Ifnar1*^−/−^ mice (*n* = 6) were infected with TOSV on the dorsal side of their left foot, either with or without the addition of TLR2 ligand Pam3CSK4 and TLR4 ligand LPS. The expression of the viral TOSV NS gene was measured using qPCR at 72hpi. Infectious units in serum were determined by plaque assay. (B) To determine if salivary microbiota affects the modulation of TOSV infection in a mammalian host, mice (*n* = 6) were inoculated on the upper side of their left foot with 100,000 PFU TOSV, either alone or in combination with 2–4 sand fly bites, which were either untreated or pre-antibiotic treated. Antibiotics included a cocktail of penicillin, streptomycin and gentamicin sulfate. The expression of the viral TOSV NS gene was measured using qPCR at 72hpi. Infectious units in serum were determined by plaque assay. (C) To determine which class of molecule within sand fly saliva was responsible for enhancing virus infection we subjected preparations of SGE (n, normal) to either heat for 10 min at 95^o^C to denature proteins (h, heat treated) or to RNAse A and RNAse T1 at 37^o^C for 20 min to remove all RNA (r, RNAase treated). *Ifnar1*^−/−^ mice (*n* = 6) were infected with TOSV on the dorsal side of their left foot, either with or without the SGE preparations. Expression of the viral TOSV NS gene was measured by qPCR at 72hpi. Plots show the median value ± interquartile range. ns = not significant, significant ∗*p* < 0.05, ∗∗*p* < 0.01, ∗∗∗*p* < 0.001, ∗∗∗∗*p* < 0.0001. Ordinary one-way ANOVA were performed for comparisons between more than two groups of normally distributed data, whereas unpaired, two-tailed Student’s *t* test were performed for comparisons between two groups.

Sand fly saliva is composed of a mixture of fly genome-encoded factors that could putatively modulate host susceptibility to virus.^25^ In addition, host response to midgut-originating microbiota following biting with *Leishmania*-infected sand flies can influence susceptibility to these parasites.^26^ Therefore, we firstly sought to define whether microbiota deposited during sand fly biting contributes to the pro-viral effects of sand fly bites. Indeed, we found the experimental spiking of TOSV inoculum with prototypic markers of bacteria (LPS and PAM3CSK4) led to significantly enhanced infection (Figure 2A). To more directly assess the effects of microbiota, we exposed mice to bites from abiotic sand flies, which had been treated with a mixture of broad-acting antibiotics (abx) that eliminate microbiota.^27^ Here, abx-treated sand flies had similar ability to enhance infection as bacteria-containing sand flies (Figure 2B). In addition, our SGE preparations had only background quantities of endotoxin at 0.02 ng/μL, which was 50,000-fold lower than the amount of LPS administered to mice (Figure 2A). So, while inclusion of high quantities of pro-inflammatory bacterial components can enhance host susceptibility to TOSV, this is likely a phenocopy, and that fly genome-encoded factors are likely responsible.

Fly genome-derived factors include, proteins, peptides, lipids, and small RNAs, all of which could putatively be responsible for enhancing TOSV infection. To define which of these classes of molecule within sand fly saliva was involved, we subjected preparations of SGE to either heat treatment for 10 min at 95^°^C to denature proteins, or combined RNAse A and RNAse T1 treatment to remove all RNA. Infection of mice with TOSV on with these SGE preparations resulted in differing quantities of TOSV RNA by 72 hpi (Figure 2C). Interestingly, heat inactivation had little impact on the ability of SGE to promote TOSV infection, while RNAse-treated SGE was less potent in its ability to enhance virus.

### Sand fly SGE worsens clinical outcome to TOSV infection

**Figure 3:**
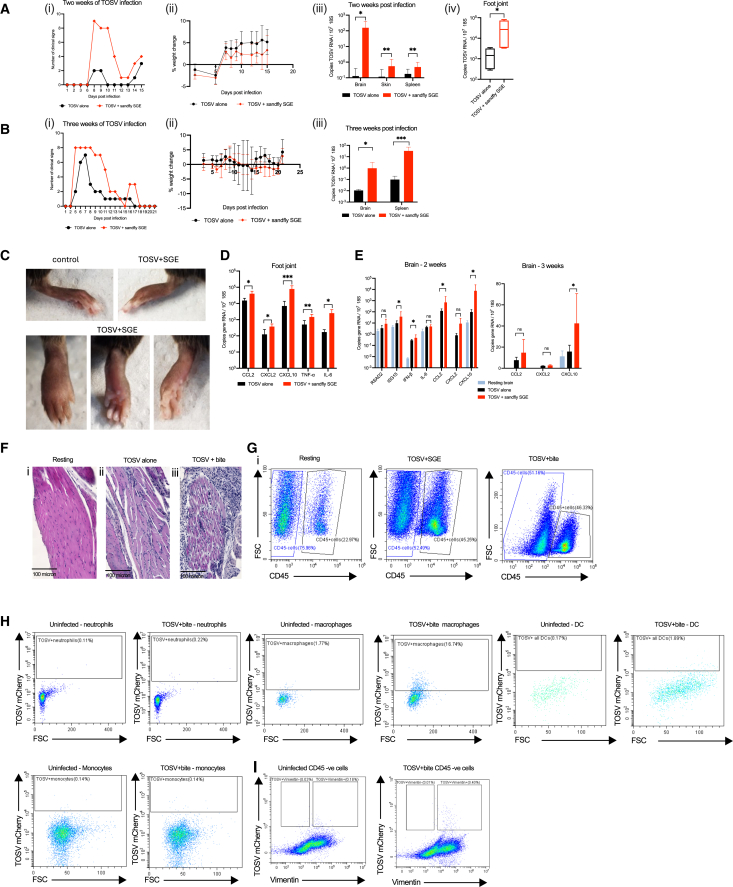
SGE enhances severity of clinical outcome in mice (A and B) *Ifnar1*^−/−^ mice (*n* = 10) were infected with 10^5^ PFU TOSV with or without *P*. *perniciosus* SGE and observed for development of clinical signs (i), and change in weight (ii), for either 2 weeks (A) or separately for 3 weeks (B). Tissues were sampled at the end of each experiment and TOSV RNA quantified by qPCR (iii). (iv) Separately, at 7 d post infection, whole foot limb with skin removed, homogenized and TOSV RNA quantified by qPCR (*n* = 5). (C) Typical gross pathological observation of swollen feet at 7 days post infection with TOSV infection with SGE. An uninfected mouse with non-inflamed foot is shown for control. (D) *Ifnar1*^−/−^ mice (*n* = 5) were infected with 10^5^ PFU TOSV with or without *P*. *perniciosus* SGE and proximal whole joint/limb sampled at day 7. Gene transcripts were quantified by qPCR. (E) *Ifnar1*^−/−^ mice (*n* = 10) were infected with 10^5^ PFU TOSV with or without *P*. *perniciosus* SGE. 2- or 3-week post infection, mice were culled, left brain hemisphere sampled and gene transcripts quantified by qPCR. (F and G) *Ifnar1*^−/−^ mice were injected with 10^5^ PFU TOSV with or without *P*. *perniciosus* SGE, or injected into *Lu*. *Longipalpis* bitten skin and at day 6 post infection, foot joints were either decalcified and stained for hematoxylin and eosin (F) or single cell solution generated for flow cytometry (G). Shown are representative sections foot musculature (F), with scale bars representing 100 microns and flow cytometry plots (G). (H and I) *Ifnar1*^−/−^ mice were injected with 10^5^ PFU mCherry-TOSV into resting or *Lu*. *Longipalpis* bitten skin, and at day 6 post infection, foot joint connective tissues dissociated into single cells and stained for flow cytometry. Gating used to define the population of TOSV+ cells was defined using fluorescence minus one (FMO) controls that lacked infection with TOSVmCherry. Shown are representative plots. All graph plots show the median value ± interquartile range. Significant ∗*p* < 0.05, ∗∗*p* < 0.01, ∗∗∗*p* < 0.001. Kruskal-Wallis test with Dunn’s multiple comparison test were used for comparisons between more than two groups, whereas non-parametric Mann-Whitney were performed for comparisons between two groups.

TOSV infection in humans most typically presents as a febrile illness in which myalgia and arthralgia are common symptoms. A minority of patients also progress to develop neurological signs with meningitis and encephalitis.^1^^,^^5^ To establish whether the inclusion of sand fly SGE could also modulate the development of clinical outcomes to TOSV infection in mice, we observed mice for up to three weeks post infection (Figures 3A–3C). Mice inoculated with TOSV in presence of SGE developed swollen feet in the joint proximal to inoculation site, peaking at days 7–11 post-infection, after which swelling slowly resolved (Figure 3C). This contrasted with TOSV infected mice inoculated without SGE, in which joint swelling was uncommon and more rapidly resolved (Figures 3Ai and 3Bi). Limbs and joints distal from the inoculation site did not exhibit notable signs of swelling, and mice were able to feed as normal; nonetheless, mice receiving SGE with TOSV gained weight less efficiently (Figures 3Aii and 3Bii). A small but notable number of mice inoculated with TOSV with SGE also developed neurological signs at later stages of infection from day 14–18. These signs included confusion and atypical repetitive paw movements (Figures 3Ai and 3Bi). Importantly, clinical signs were associated with a more efficient dissemination of virus to these sites, as assessed by quantification of virus RNA at the end of each clinical observation. Virus RNA was significantly higher in skin, spleen, and brain tissue in mice that had received SGE (Figures 3Aiii and 3Biii). We also found virus RNA was more highly expressed in feet joint/musculature of SGE-recipient mice at day 7 post infection, at the time of peak limb swelling (Figure 3Aiv). Together these suggest early events at the skin inoculation site, in which SGE boosts early TOSV replication, and enables more efficient dissemination of virus to proximal connective tissue and the brain. SGE-enhanced dissemination of virus to joint tissues resulted in higher upregulation of key arthritogenic cytokine transcripts, including TNF-alpha and IL-6, and the chemokines CXCL2, CCL2, and CXCL10 at day 7 post infection (Figure 3D). Similarly, brain tissue sampled from the mice with neurological signs had higher expression of key encephalitic mediators, most notably the chemokine CXCL10 (Figure 3E).

Foot swelling was associated with pathological changes to musculature tissue (Figure 3F). SGE-enhanced TOSV infection resulted in gaps forming between muscle fibers, indicative of subcutaneous edema and myofiber degeneration and an influx of mononuclear and polymononuclear cells in the subcutaneous tissue, extending into the muscular layer. This leukocyte influx was also evident upon flow cytometry of connective tissue cells in mice receiving SGE in the TOSV inoculum, or in mice infected with TOSV at sand fly bitten skin (Figure 3G). To determine whether connective tissue cells become infected with TOSV, we infected mice with an engineered form of the virus that expressed mCherry. We firstly determined that this strain of TOSV was similarly sensitive to sand fly SGE-mediated enhancement of the infection (Figure S2C). Using the gating strategy depicted in Figures S2A and S2B, we found most joint/connective tissue inflammatory leukocytes including neutrophils, monocytes, and dendritic cells (DCs) exhibited low to none mCherry expression (Figure 3H). In comparison, a notable number of MerTK+ve macrophages were positive for mCherry. Within the CD45-ve fraction, the only mCherry+ve cells were also positive for vimentin, indicative of fibroblast infection (Figure 3I). Together these data show co-inoculation of TOSV with sand fly SGE, or inoculation of TOSV into sand fly-bitten skin, enhanced dissemination of virus to other tissues and the development of clinical signs that are commonly reported with human infection. In feet joint tissues, virus infected both macrophages and fibroblasts, activating pro-inflammatory gene expression and worsened histopathology.

### Skin inflammatory gene expression to TOSV infection is enhanced by sand fly saliva

We hypothesized that SGE modulates host susceptibility through action at the skin inoculation site. We firstly assessed whether sand fly SGE could directly modulate cell susceptibility to virus by infecting primary cultures of skin fibroblasts, macrophages, and DCs *in vitro*. Here, the addition of SGE to the virus inoculum did not modulate the ability of virus to replicate in these cultures (Figure S3A), suggesting the mechanism was dependent on processes occurring *in vivo*. Since SGE-mediated enhancement of infection was independent of type I IFN signaling, we instead hypothesized that SGE may suppress the generation of virus neutralizing antibodies. However, serum-neutralizing antibody quantities were similar in mice irrespective of SGE inclusion with inoculum, and indeed were slighted elevated in mice receiving SGE, perhaps reflecting higher virus titers in these mice (Figure S3B).

Next, because macrophages were positive for TOSV-mCherry in infected joints (Figure 3H), we hypothesized that SGE and/or sand fly biting may enhance infection through recruitment of these leukocytes to skin. We thought this to be likely as mosquito saliva, although distinct to sand fly saliva, can similarly enhance skin infection with virus by promoting entry of virus-permissive leukocytes that efficiently replicate virus.^5^^,^^16^^,^^28^ We firstly defined whether SGE or sand fly biting modulated the expression of key pro-inflammatory cytokines and chemokines that mediate myeloid cell recruitment to skin. In wild type mice, by 24 hpi with either SFV or TOSV, the inclusion of SGE caused a trend in increased expression of inflammatory chemokine (*ccl2* and *cxcl2*) and IFN-responsive gene transcripts (*isg15* and *rsad2*), although this only reached statistical significance for IL-6 (Figure S4A). In the draining LN, all inflammatory genes assessed were more highly expressed in SGE recipients. In *ifnar1*-null mice, those that also received SGE with virus exhibited only modest increases in inflammatory gene expression (Figures S4B–S4D). However, mice receiving a sand fly bite exhibited significantly higher expression of skin *ccl2*, *cxcl2*, *ISG15*, and *IL-6* compared to mice that had received virus alone (Figure S4D).

### Sand fly saliva-recruited macrophages become infected with TOSV but do not release infectious virus

**Figure 4:**
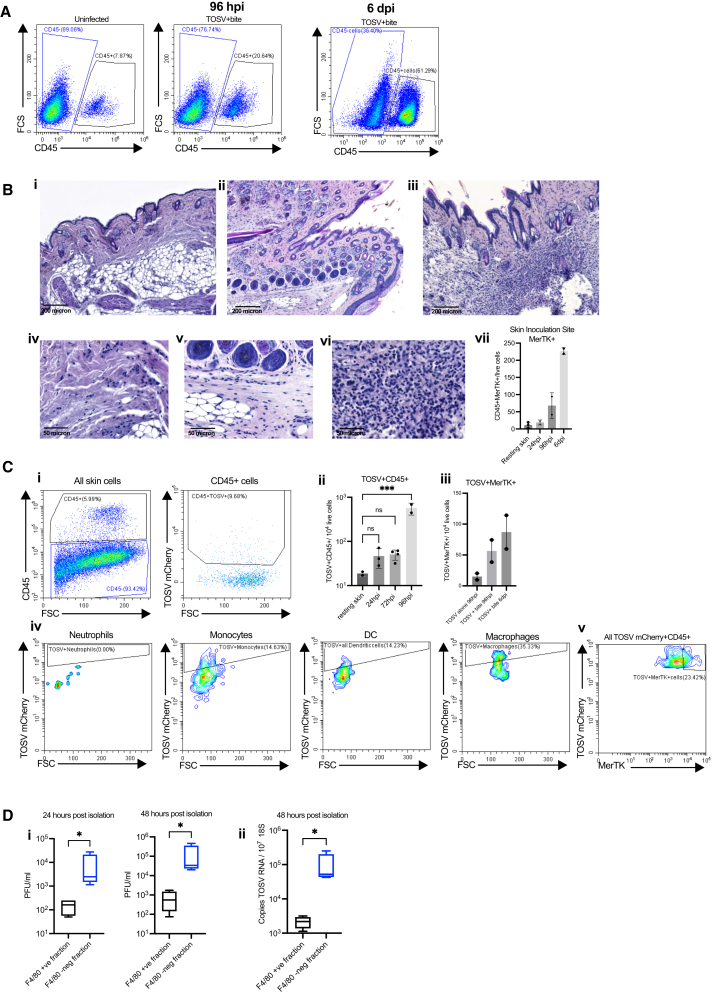
Sand fly SGE recruits macrophages that become infected with TOSV, but do not release infectious virus (A) *Ifnar1*^−/−^ mice were either left uninfected or exposed to *Lu*. *longipalpis* sand fly bites and immediately infected with 10^5^ PFU TOSV-mCherry. At times indicated post infection, skin cell were stained for flow cytometry. (B and C) *Ifnar1*^−/−^ mice were infected with 10^5^ PFU TOSV with or without *P*. *perniciosus* SGE. (B) At 6 days post infection foot skin (upper, dorsal side) stained for hematoxylin and eosin. Shown are representative images of whole skin, and higher magnification of lower dermis for mice receiving (i and iv) saline control, (ii and v) TOSV, (iii and vi) and TOSV and SGE, skin. Scale bars represent either 200 microns (i–iii) or 50 microns (iv–vi). (vii) Skin cells were stained for flow cytometry and gated on live CD45^+^CD11b+MerTK+FSC^hi^ to define macrophage frequency. (C) At 24 hpi to 96 hpi, skin cells were stained for flow cytometry and gated on leukocyte cell type specific gates to define mCherry expression. (i) representative gates for CD45 gating at 72 hpi; (ii) quantification of mCherry+CD45^+^ cells; (iii) quantification of mCherry+CD45^+^CD11b+MerTK+FSC^hi^ cells; (iv) representative plots showing mCherry expression in defined leukocyte cell types; (v) representative plot showing all mCherry+ cells against macrophage MerTK expression. Gating used to define the population of TOSV+ cells was defined using fluorescence minus one (FMO) controls that lacked infection with TOSVmCherry. Bars represent mean ± SD. ns = not significant, significant ∗*p* < 0.05, ∗∗∗*p* < 0.001, using ordinary one-way ANOVA. (D) *Ifnar1*^−/−^ mice were infected with 10^5^ PFU TOSV with *P*. *perniciosus* SGE and skin F4/80 macrophages isolated using magnetic beads. Macrophages were cultured separately to remaining macrophage depleted skin cells, and infectious virus released to tissue culture supernatant quantified by plaque assay. Graph plots represent the median value ± interquartile range. Significant ∗*p* < 0.05, using Mann-Whitney.

To define whether increased chemokine expression was associated with more extensive leukocyte recruitment to skin, we assessed the number of CD45^+^ cells by flow cytometry and found an increase in leukocyte numbers by day 4 post bite, that was further elevated by day 6 (Figure 4A). Needle administration of SGE alone was also sufficient to recruit and retain leukocytes to the skin, as shown by flow cytometry at day 3 (Figure S5) and histology by day 7 (Figure 4B). SGE-administered skin at day 7 exhibited enlarged dermis, with numerous monocytic cells in the lower dermis, compared to resting skin and mice receiving virus alone. Further assessment by flow cytometry revealed increased frequency of SGE-recruited macrophages (CD45^+^, CD11b^+^, MerTK^+^, and Ly6G^-^) by 96 h post injection. To define whether these infiltrating leukocytes become infected with virus we infected skin with mCherry-expressing TOSV^29^ with SGE and comprehensibly assessed the ability of TOSV to infect key leukocyte cell types (as gated in Figures S2A and S2B). Initial assessment of CD45^+^ leukocytes showed some were TOSV-mCherry+ve (Figure 4Cii), and that this frequency increased over time (Figure 4Cii) and included mCherry+ve macrophages (Figure 4Ciii). Further dissection of the leukocyte gate revealed neutrophils were negative for TOSV-mCherry, while DC and monocyte gates had a low frequency for being mCherry+ve. In contrast, over a third of all events in the macrophage gate were mCherry+ve (Figure 4Civ). Infected MerTK+ve cells represented almost a quarter of all infected skin cells (Figure 4Cv).

mCherry is encoded by TOSV, and its expression is indicative of infection. However, it was also possible that these cells gained fluorescence through phagocytosis of infected cells or could represent cells that were infected but not releasing infectious virus. Therefore, we isolated macrophages from the TOSV-infected skin and cultured them *ex vivo* and quantified the amount of infectious virus released at 24–48 h in culture. Surprisingly, the macrophage fraction released little infectious virus, while the macrophage negative fraction, representing all other skin cell types, generated 54-fold more virus (Figure 4D). Therefore, although macrophage frequency was increased by SGE, and became positive for virus encoded mCherry, they were not capable of generating new infectious virus.

### Sand fly SGE reprograms skin fibroblasts to become more primitive and susceptible to TOSV infection

**Figure 5:**
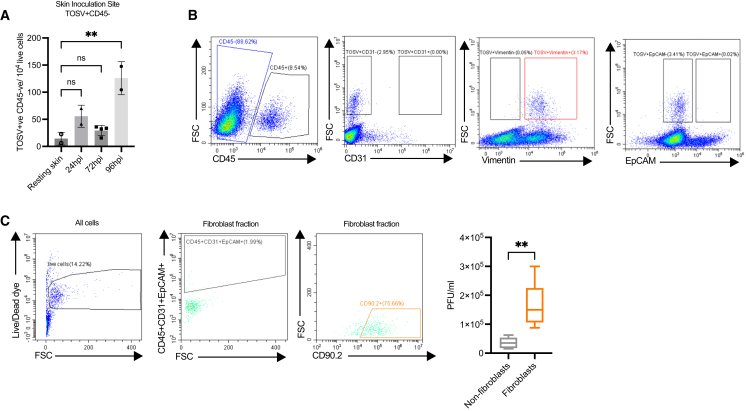
Fibroblasts become infected and generate infectious TOSV (A and B) *Ifnar1*^−/−^ mice were infected with 10^5^ PFU mCherry-TOSV and *P*. *perniciosus* SGE. (A) Skin cells were stained for flow cytometry and gated on live mCherry+CD45-ve cells to define frequency of non-hematopoietic infected cells. Bars represent mean ± SD. (B) CD45-ve cells were gated and expression of markers of cell type (CD31, endothelial; vimentin, fibroblast; EpCAM (CD326), epithelial) shown against TOSV-mCherry expression. (C) *Ifnar1*^−/−^ mice were infected with 10^5^ TOSV and *P*. *perniciosus* SGE and at 72 hpi, skin fibroblasts were isolated through one round of negative selection, followed by one round of positive selection for CD90.2 cells. Cells were cultured *ex vivo* for 24 h and infectious virus released to supernatant quantified by plaque assay (*n* = 5 mice). Graph plots represent the median value ± interquartile range. ns = not significant, significant ∗∗*p* < 0.01 using ordinary one-way ANOVA for comparisons between more than two groups and Mann Whitney for comparisons between two groups.

We next defined which cell types become infected with TOSV in the non-leukocyte fraction of skin. We found that CD45-negative cells became TOSV-mCherry+ve following infection with SGE, and that this increased over time (Figure 5A). To define which cell type was becoming infected we devised a separate flow cytometry panel (Figure S6). We found that endothelial and epithelial cells were negative for mCherry, while almost all CD45-ve mCherry+ cells were positive for the pan-fibroblast marker vimentin (Figure 5B). Isolation of fibroblasts using magnetic selection was performed to high purity (Figure 5C) to assess their ability to release new infectious virus and demonstrated that the majority of new infectious virus was generated by this cell type (Figure 5C).

**Figure 6:**
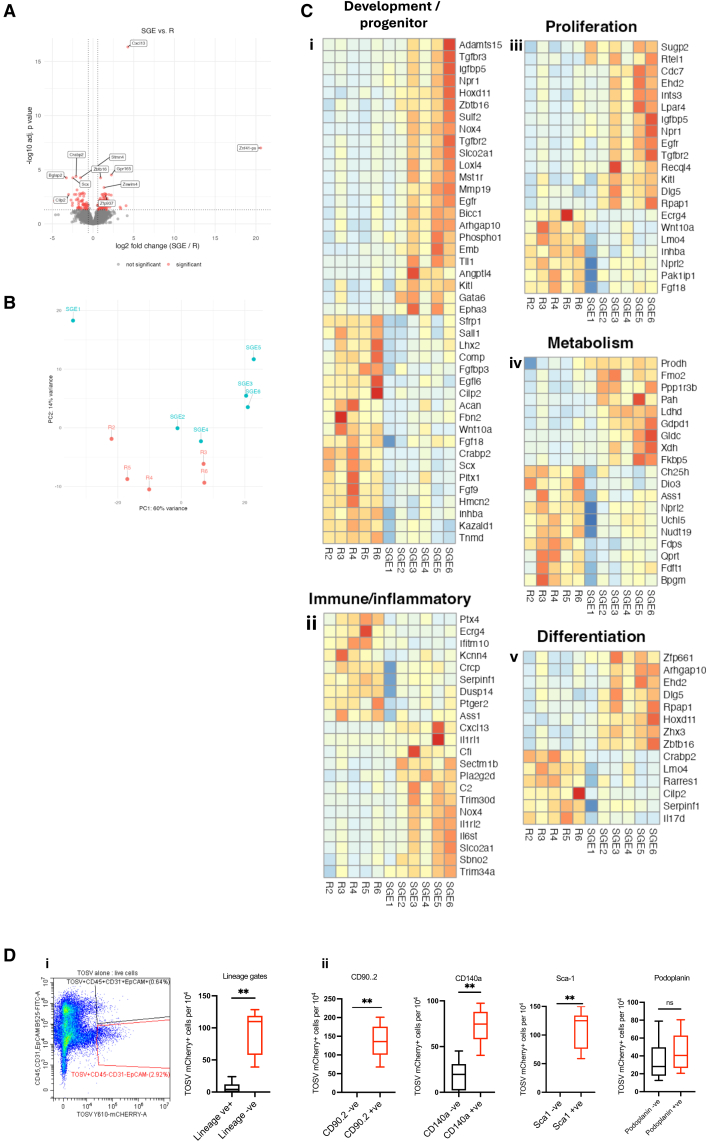
Sand fly SGE reprograms fibroblasts to enable infection of Sca1+ primitive fibroblasts (A–C) *Ifnar1*^−/−^ mouse skin (*n* = 6) was administered with either *P*. *perniciosus* SGE or saline control and at 72 h, skin fibroblasts isolated through one round of negative selection, followed by one round of positive selection for CD90.2 cells, and lyzed for RNA extraction. (A) Following RNA-seq and data processing DEG were defined (fold change > log2(1.5), adjusted *p* value < 0.05) and shown here as a volcano plot. (B) Principle component analysis to show clustering of samples. Each dot represents one biological sample (R = resting skin). Note one resting sample was removed due to poor RNA-sequence read alignment. (C) Hierarchical clustering of DEG fold change into 5 GO defined descriptors. (D) *Ifnar1*^−/−^ mouse skin (*n* = 6) was infected with mCherry-TOSV with *P*. *perniciosus* SGE and at 72 h skin cells stained for flow cytometry. (i) Single live cells were gated to define CD45, EpCAM and CD31 positive cells (lineage positive) and fibroblast containing gate (lineage negative cells). Frequency of TOSV-mCherry positive cells were quantified in each lineage. (ii) Lineage negative cells were assessed for frequency of mCherry+ cells in specific fibroblast markers. Graph plots represent the median value ± interquartile range. ns = not significant, significant ∗∗*p* < 0.01, using Mann Whitney.

Fibroblasts are a heterogenous cell type present at high frequency in the skin. They are a versatile, non-hematopoietic cell type involved in tissue homeostasis and wound repair, capable of transitioning between quiescent, activated, and differentiated states in response to environmental cues.^30^^,^^31^ Fibroblasts also partake in immunomodulatory function by responding to inflammatory signals and expressing pro-inflammatory cytokines.^32^ To assess whether sand fly saliva was modulating the biology of this cell type we administered SGE alone to mouse skin and isolated fibroblasts 72 h later. Extracted RNA was then subject to RNA-sequencing to define the type and number of differentially expressed genes (DEGs). We identified a total of 166 DEG, with 63 downregulated and 103 upregulated in fibroblasts from SGE-administered skin, compared to resting saline-injected skin (Figure 6A). Principal component analysis revealed that fibroblasts from saliva-treated skin clustered separately from resting fibroblasts, indicating a distinct transcriptional reprogramming (Figure 6B).

Gene ontology (GO) analysis revealed that the majority of DEG fell into 5 groups of descriptors: developmental/progenitor (42 DEG), proliferation (17 DEG), metabolism (19 DEG), immune/inflammatory (23 DEG), and differentiation (13 DEG). Hierarchical clustering of DEG revealed patterns of upregulated and downregulated DEGs within each of these descriptors (Figure 6C). Some of the most significantly differentially regulated developmental genes (Figure 6Ci) included those associated with either TGF-β signaling (e.g., *tgfbr2* and *tgfbr3*) or progenitor/stem cell regulation (e.g., *gata6*, *Angptl4*, *Emb*, *Loxl4*, *Mmp19*, *Npr1*, *Nox4*, *Slco2a1*, *Mst1r*, and *Epha3*). Together these progenitor genes can influence extracellular matrix remodeling associated with wound healing.^33^^,^^34^^,^^35^ Other developmental DEGs of note were upregulation of the pro-fibrotic *zbtb16* (promyelocytic leukemia zinc finger)^36^ and downregulation of *crabp2* (cellular retinoic acid-binding protein 2).^37^ Several immune DEGs (Figure 6Cii), including the second most upregulated gene *cxcl13*, along with *Il6st* and *Il1rl2*, suggests an inflammatory response to SGE. DEGs associated with fibroblast proliferation (Figure 6Ciii) included *Cdc7*, *IntS2*, *Lpar4*, *Egfr*, *Tgfbr2*, *Dlg5*, *Igfbp5*, and *Rpap1*, suggesting fibroblast transition to more primitive, active state, consistent with a wound healing or regenerative response.^38^^,^^39^^,^^40^^,^^41^ *Igfbp5* additionally also has a pro-fibrotic role.^42^ The upregulation of *ppp1r3b*, *gldc*, *xdh*, and *fkpb5* (Figure 6Civ) is notable as they are associated with metabolic reprogramming that occurs with cellular dedifferentiation, stress responses, or wound healing. Finally, several DEGs were associated with differentiation, including downregulation of *Hoxd11*, *Zfp661*, *Zhx3*, and *Zbtb16*, suggesting reduced differentiation signature, aligning with a dedifferentiation or primitive state. Indeed, a separate unsupervized analysis of all downregulated DEGs revealed that GO descriptors were dominated by developmental genes (Figure S7).

Together these show that sand fly saliva reprogrammed skin fibroblasts either directly or indirectly (e.g., via enhanced inflammatory response; Figures 4 and S4) to adopt a more primitive state, putatively to restore tissue integrity following the SGE-mediated inflammatory challenge. Cells that are developmentally more immature/less specialized often have a higher capacity for proliferation and are typically more susceptible to arbovirus infection than differentiated cells.^43^^,^^44^^,^^45^ Therefore, we hypothesized that TOSV may replicate more efficiently in fibroblasts from SGE-treated skin due to their more primitive nature.

To determine whether TOSV preferentially replicated in more primitive fibroblasts, we assessed differentiation and activation marker expression of TOSV-infected fibroblasts in skin. We first labeled most other cell types into the FITC channel (CD45, EpCAM, and CD31), which was referred to as lineage positive. The remaining cells were referred to as lineage negative and contained mainly fibroblasts (Figures 6D and S6). Using this approach, we could directly define the number of infected cells in each lineage and found the majority of mCherry+ve cells were in the fibroblast (lineage negative) gate (Figure 6Di). Within the lineage-negative gate, we found that almost all the TOSV-mCherry+ve cells were positive for CD90.2 and CD140a, confirming their status as fibroblast cells (Figure 6Dii). Importantly, TOSV-mCherry +ve cells were exclusively positive for the progenitor pluripotent mesenchymal marker, stem cell antigen 1 (Sca1). Sca1 marks the identity of a fibroblast progenitor subpopulation in the lower, reticular dermis that has enhanced plasticity, self-renewal capacity, and regenerative potential.^46^^,^^47^ Most infected fibroblasts were also positive for podoplanin, a marker of fibroblast activation.^48^ Together these suggest that TOSV preferentially infects fibroblasts that are activated or in a progenitor-like state.

To test whether fibroblast reprogramming was a direct effect of SGE exposure or driven indirectly by inflammation and leukocyte recruitment, we stimulated fibroblasts *in vitro* with filter-sterilized SGE under sterile, serum-low conditions (Figure S8). Fibroblasts were cultured either alone or with macrophages (1:5 ratio) to assess whether macrophage-derived inflammatory factors could also induce reprogramming. However, expression of three exemplar genes from the RNA-sequencing dataset (*tgfrb2*, *gata6*, and *zbtb16*), representing transcriptional regulators and signaling components of fibroblast differentiation, was unchanged under either condition. These findings indicate that SGE-mediated fibroblast reprogramming is exclusively observed following an *in vivo* specific process.

## Discussion

This study identifies sand fly saliva as a critical modulator of arboviral pathogenesis, demonstrating that salivary factors from two distinct sand fly genera significantly enhance infection with both TOSV and the unrelated SFV. While this enhancement was associated with the recruitment and infection of dermal macrophages, these cells produced little infectious virus. Instead, sand fly saliva reprogrammed fibroblasts into a wound-healing state, which appeared more permissive to viral replication. These events not only amplified skin viral replication but also promoted systemic dissemination and the onset of clinical disease, including arthritis-like and neurological signs. Furthermore, to our knowledge, this is the first TOSV mouse model to recapitulate key neurological features of human disease following extraneural inoculation.^49^^,^^50^^,^^51^ Our finding that sand fly saliva significantly enhances TOSV replication and dissemination, even in semi-permissive mice, suggests that vector-mediated modulation may help overcome species-specific barriers, with implications for arboviral host range and zoonotic potential.

We establish that sand fly saliva is not merely a passive vehicle for virus delivery but an active modulator of the skin environment, shaping the outcome of infection. SGE-induced reprogramming of fibroblasts into a more primitive state drove both local and systemic infection. Importantly, this effect was conserved across two taxonomically unrelated viruses, TOSV (a phlebovirus within *Bunyaviricetes*) and SFV (an alphavirus within *Togaviridae*), as well as across distinct sand fly species, indicating that saliva-induced modulation can act broadly across divergent viral families. Consistent with this, our analyses identified fibroblasts as the principal non-leukocyte target of infection and the dominant contributors to saliva-enhanced replication.

The ability of sand fly saliva to enhance infection with genetically divergent arboviruses implies a conserved vector-mediated strategy on host processes, possibly via shared effects on early skin-resident cells.^12^ Fibroblasts were the only non-leukocyte population in which we detected infection, highlighting them as principal drivers of saliva-enhanced replication. This is somewhat surprising, as TOSV infection of endothelial cells has been reported,^52^ although this may reflect secondary spread to these cells via the bloodstream. These findings redefine fibroblasts not only as structural or immunomodulatory cells, but as key targets for early arbovirus replication, whose susceptibility is shaped by the vector saliva. While macrophages were frequently mCherry+ve, the absence of detectable infectious virus suggests either abortive infection or phagocytosis of infected material. It is possible that mCherry signal reflected intrinsic restriction, as described for myeloid cells in other arboviral infections, where virus entry occurs but replication is blocked. While macrophages were frequently mCherry+ve, the absence of detectable infectious virus suggests either abortive infection, phagocytosis of infected material, or restriction of viral replication. Similar outcomes have been described in myeloid cells during other arboviral infections, where infection is initiated but productive replication is limited or absent. For example, ZIKV and DENV can enter macrophages yet replicate inefficiently,^53^^,^^54^ and WNV replication in macrophages is often constrained by innate antiviral mechanisms.^55^ These parallels suggest that macrophages may act more as virus reservoirs or sinks rather than producers, in contrast to fibroblasts which we identified as the principal source of infectious virus. This highlights the need for caution when interpreting reporter signals as evidence of productive replication. Having established fibroblasts as the primary cellular drivers of saliva-enhanced infection, we next compared how closely our SGE model reflected natural sand fly biting.^53^^,^^54^^,^^55^

The outcomes with needle-injected SGE were also observed with natural sand fly biting, where enhancement was even stronger (Figure 1F). Viremia was detectable in sand fly-bitten mice, while TOSV RNA quantities were approximately two times higher. The greater enhancement observed following biting compared with SGE injection likely reflects not only the delivery of salivary factors but also the unique microenvironment created by vector feeding, including localized tissue trauma, vascular leakage, and highly focal deposition of saliva. Furthermore, as our SGE preparations were derived from just one salivary gland, it is possible that biting delivers a higher quantity of saliva than that achieved by needle injection. Finally, SGE preparations can vary from true saliva in protein concentration, delivery kinetics, and potential contamination with intracellular material that might be released during gland dissection. Thus, while SGE injection provides a tractable and reductionist approach to dissect underlying mechanisms, the live-bite data reinforce the physiological relevance of our model and demonstrate that these results are not artifacts of SGE administration. More broadly, the greater enhancement of infection following natural sand fly biting underscores the importance of salivary delivery dynamics, including microtrauma, vascular leakage, and highly localized cell activation, which are difficult to replicate by needle inoculation.^13^

These findings, together with previous work on other arthropod vectors, situate our study within a wider framework of how saliva from blood-feeding insects shapes pathogen transmission. As such, these new findings build on a broader body of work demonstrating that saliva from blood-feeding arthropods can significantly shape host susceptibility to infection. In the context of *Leishmania*, seminal studies^20^^,^^22^^,^^56^ showed that sand fly saliva exacerbates disease by recruiting leishmania-permissive cells such as macrophages and neutrophils.^21^ Similar principles have been observed for mosquito-borne viruses, where *Aedes* and *Culex* mosquito saliva has both been shown to skew early innate immune responses, disrupt endothelial barriers, and enhance viral replication.^16^^,^^57^^,^^58^ Our study extends these concepts by identifying fibroblasts as critical and previously overlooked responders to sand fly saliva. While prior studies emphasized immunomodulation or leukocyte recruitment, our data reveal that salivary factors actively reprogram the skin’s stromal compartment, potentially creating a regenerative niche that favors viral replication. As such, the interplay between stromal cells and immune modulation by vector saliva at the inoculation site are central to the pathogen establishment and dissemination. An important next step is to move from cellular outcomes to the underlying salivary factors responsible.

Although we likely excluded a role for microbial components in sand fly bites, the specific salivary molecules responsible for fibroblast reprogramming remain unidentified. Several immunomodulatory proteins are well characterized in *Phlebotomus* saliva, including maxadilan, adenosine deaminase, apyrases, and D7-like proteins, each of which alters host vascular biology, platelet aggregation, or inflammatory responses.^25^ Whether such proteins directly influence fibroblast biology is unknown, but their presence highlights the molecular complexity of saliva and potential for multiple salivary factors to act in concert. Crucially, we show that one of these factors within SGE is a heat-stable RNAse-sensitive class of molecule, putatively implicating small RNAs. These data also suggest that complex heat-sensitive, protein-based molecules are unlikely required. The role for lipid-based molecules was not assessed.

In summary, while our model recapitulated systemic disease features, including neurological signs, the mechanistic links between early skin events and distal pathology remain to be defined. Future studies using recombinant salivary factors and fibroblast-targeted interventions will be crucial to elucidate these pathways. Together, our findings identify a key role for sand fly saliva in arbovirus transmission and pathogenesis, highlighting the skin as a dynamic inflammatory niche where vector-derived factors shape the disease trajectory.

### Limitations of the study

While this study provides novel insights into how sand fly saliva modulates host susceptibility to arboviral infection, several limitations remain.

#### Use of IFNAR1^−/−^ mice

A limitation of our TOSV model is the reliance on IFNAR1^−/−^ mice, which, while necessary to permit infection, constrains the interpretation of immune mechanisms, particularly those dependent on type I IFN. However, our demonstration that SFV infection was also enhanced by SGE in wild-type immunocompetent mice provides strong support that the phenomenon we describe is not simply an artifact of the IFN-deficient model. This cross-validation strengthens the conclusion that saliva acts as a general modulator of infection rather than a virus-specific phenomenon.

#### Generalizability across phleboviruses

While we show that saliva enhances infection with both TOSV and the unrelated SFV, it remains to be tested whether similar enhancement occurs with other sand fly-borne phleboviruses, such as Sandfly Fever Naples virus. Future studies could assess whether fibroblast reprogramming is a broadly applicable mechanism across diverse arboviruses.

#### TOSV-mCherry expression in macrophages

The frequent detection of mCherry in macrophages, despite minimal infectious virus production, raises uncertainty about whether these cells are productively infected, phagocytosing infected material, or experiencing restricted replication. These findings align with previous reports of abortive infection in myeloid cells during arboviral infection and warrant cautious interpretation of reporter signal.

#### Identify of pro-viral factors in sand fly saliva

Although our data implicate a heat-stable, RNase-sensitive component in fibroblast reprogramming, the precise salivary molecule remains unknown. While known immunomodulatory proteins are present in *Phlebotomus* saliva, further work is needed to determine their relevance to fibroblast function and viral enhancement.

## Resource availability

### Lead contact

Requests for further information and resources should be directed to and will be fulfilled by the lead contact, Clive S McKimmie (clive.mckimmie@york.ac.uk).

### Materials availability

This study did not generate new unique reagents.

### Data and code availability

• Data reported in this paper will be shared by the lead contact upon request. All RNA-Sequence data have been deposited in a publicly available database, as described in the key resources table.
• This paper does not report original code.
• Any additional information required to reanalyze the data reported in this paper is available from the lead contact upon request.

## Acknowledgments

We gratefully acknowledge the Leeds Biomedical Services and York Biological Services for technical assistance with mouse procedures and the Leeds Faculty of Medicine & Health Flow Cytometry Facility for their support. The authors thank Dr Sally James of the Genomics Laboratory at the University of York Bioscience Technology Facility for their support and assistance in this work. The authors gratefully acknowledge Dr Peter O’Toole, Karen Hogg, and Graeme Park of the Bioscience Technology Facility at the University of York for their support and assistance with flow cytometry. The authors gratefully acknowledge Dr Alastair Droop and the Genomics and Bioinformatics Laboratory, University of York. This work was funded by a scholarship to Y.K.T. from Republic of Türkiye Ministry of National Education for a Study Abroad Postgraduate Education Scholarship (MEB1416) and a 10.13039/100009001University of York startup grant. C.S.M. and A.M.-B. were funded by the MRC Medical Research Council (MR/N013840/1). M.G.C. was funded by the EU-MUR PNRR Extended Partnership (PE00000007, CUP B63C22001400007, INF-ACT), Italian MUR PNR (D.M. 737). A.K. was funded by EU Infravec2 (No 731060) and the UK MRC (MC_UU_12014/8, MC_UU_00034/4). M.E.R. was funded from the UK MRC VALIDATE program (MR/R005850/1). P.V. and M.J. were supported by a research project funded by the project National Institute of Virology and Bacteriology (program EXCELES, ID project no. LX22NPO5103), funded by the 10.13039/501100000780European Union – Next Generation EU.

## Author contributions

Conceptualization, Y.K.T., K.S., and C.S.M.; data curation, Y.K.T., S.M., and C.S.M.; formal analysis, Y.K.T., S.M., and C.S.M.; funding acquisition, Y.K.T., P.V., and C.S.M.; investigation, Y.K.T., A.M.-B., L.B., M.J., M.E.R., A.J.T.A., C.K., S.M., and C.S.M.; methodology, Y.K.T. and C.S.M.; project administration, Y.K.T. and C.S.M.; resources, M.J., P.V., M.E.R., A.J.T.A., M.G.C., and A.K.; supervision; P.V., M.G.C., A.K., and C.S.M.; visualization, S.M.; writing – original draft, Y.K.T. and C.S.M.; writing – review & editing, Y.K.T., A.M.-B., L.B., M.J., P.V., M.E.R., A.J.T.A., C.K., S.M., M.G.C., A.K., K.S., and C.S.M.

## Declaration of interests

The authors have no conflicts of interest to declare.

## STAR★Methods

### Key resources table

**Table:** 

REAGENT or RESOURCE	SOURCE	IDENTIFIER
**Antibodies**		

FITC-CD45	BioLegend	30-F11; Cat#103107; RRID: AB_312972
APC-CD11b	BioLegend	M1/70; Cat#101211; RRID: AB_312795
APC-Cy7-MERTK (Mer)	BioLegend	2B10C42; Cat#151519; RRID: AB_2876507
Brilliant Violet 421-Ly-6G	BioLegend	1A8; Cat#127627; RRID: AB_2562567
PE-Ly-6C	BioLegend	HK1.4; Cat#128007; RRID: AB_1186133
APC-MHC II	BioLegend	M5/114.15.2; Cat#107613; RRID: AB_313328
Pe-Cy7-CD11c	BioLegend	N418; Cat#117317; RRID: AB_493569
APC-Vimentin	Biotechne	280618; Cat#IC2105A; RRID: AB_3654983
FITC-CD326 (Ep-CAM)	BioLegend	G8.8; Cat#118208; RRID: AB_1134107
PE-CD31	BioLegend	390; Cat#102407; RRID: AB_312903
Pacific Blue-CD90.2 (Thy1.2)	BioLegend	30-H12; Cat#105323; RRID: AB_1877204
Alexa Fluor 647-Podoplanin	BioLegend	PMab-1; Cat#156203; RRID: AB_2750403
PE/Cy7-CD140a (PDGFR-α)	BioLegend	APA5; Cat#135911; RRID: AB_2715973
PE-Ly-6A/E (Sca-1)	BioLegend	W18174A; Cat#160905; RRID: AB_2910334
Zombie UV	BioLegend	RUO; Cat#423107

**Bacterial and virus strains**		

Semliki Forest Virus 4 (SFV4)	Generated by Lefteri et al.,^18^	N/A
Toscana Virus; strain 1812	Isolated from patient (Cusi et al.^23^)	N/A
Toscana Virus; strain 1500590; lineage A	Alexander et al.^29^	N/A

**Biological samples**		

C57BL/6 and *ifnar1-/-* mice skin, spleen, lymph node, brain and foot tissues, and blood serum	This paper	N/A

**Chemicals, peptides, and recombinant proteins**		

Flt3-Ligand	PeproTech/Gibco	Cat#17820733
M-CSF	PeproTech/Gibco	Cat#17822333
InVivoMab anti-mouse IFNAR-I; MAR1-5A3	BioXCell	Cat#BE0241
Streptomycin/penicillin	Gibco	Cat#15140122
GlutaMAX	Gibco	Cat#35050061
Tryptose Phosphate Broth	Gibco	Cat#18050039
Hanks balanced saline solution	Sigma-Aldrich	Cat#H9394
Collagenase P	Roche	Cat#11 213 857 001
Dispase II	Roche	Cat#04942078001
DNase I	Roche	Cat#10104159001
PerfeCTa SYBR® Green FastMix	Quantabio	Cat#95072-250
Fetal bovine serum	Sigma Aldrich	Cat#F9665-500ML
RNAlater solution	Sigma Aldrich	Cat#R0901-500ML
TRIzol	Invitrogen	Cat#15596026
2X MEM (Temin's modification)	Gibco	Cat#11935046

**Critical commercial assays**		

ToxinSensor™ Chromogenic LAL Endotoxin Assay Kit	GenScript	Cat#L00350
Tumor-Associated Fibroblast Isolation Kit, mouse	Miltenyi Biotec	Cat#130-116-474
QIAquick PCR Purification Kit	Qiagen	Cat#28106
High-Capacity RNA-to-cDNA Kit	Applied Biosystems	Cat#4387406
PureLink RNA Mini Extraction Kit	Invitrogen	Cat#12183018A
PureLink™ RNA Micro Scale Kit	Invitrogen	Cat#12183016
Cytofix/Cytoperm Fixation/Permeabilization Kit	BD	Cat#554714

**Deposited data**		

All RNASeq data in this paper deposited with this accession number.	Genbank	GSE297255

**Experimental models: Cell lines**		

Baby Hamster Kidney-21	Derived from Lefteri et al.,^18^	N/A
Vero cells	Derived from Lefteri et al.,^18^	N/A

**Experimental models: Organisms/strains**		

B6(Cg)-*Ifnar1*tm1.2Ees/J mice Strain #:028288	Jackson Laboratory	RRID:IMSR_JAX:028288
C57BL/6 mice, derived from Strain #:000664	Jackson Laboratory, bred at the University of Leeds, Animal house	JAX: 000664

**Oligonucleotides**		

See Table Table S1 for all sequences		

**Statistical section**		

Ordinary-one-way ANOVA was performed for comparisons between more than two groups of normally distributed data.		
Unpaired, two-tail Student’s t test was performed for comparisons between two groups.		
Kruskal-Wallis test with Dunn’s multiple comparison test was used for comparisons between more than two groups, whereas non-parametric Mann-Whitney was performed for comparisons between two groups where data had non-Gaussian distribution		
In all cases n = number of mice used		
All plots have statistical significance indicated with ∗p < 0.05, ∗∗p < 0.01, ∗∗∗p < 0.001, ∗∗∗∗p < 0.0001, ns = not significant		
Statistical details of all experiments can be found in the figure legends and STAR Methods section.		
Whisker plots represent median average +/- interquartile range		
Column plots represent mean +/- SD		

**Software and algorithms**		

CytExpert software; CytoFLEX Platform	Beckman Coulter	N/A
Graph Pad Prism version 10	Graph Pad Software	https://www.graphpad.com/

**Other**		

MS Columns	Miltenyi Biotec	Cat#130-042-201
LD Columns	Miltenyi Biotec	Cat#130-042-901
QIAshredder column	Qiagen	Cat#79656
Anti-F4/80 MicroBeads UltraPure, mouse	Miltenyi Biotec	Cat#130-110-443
Anti-Rat/Hamster Ig κ/Negative control compensation beads	BD	Cat#552845
Anti-Mouse CD16/CD32 (Mouse BD Fc Block)	BD Pharmingen	Cat#553141
Stainless-steel beads 7mm	Qiagen	Cat#69990

### Experimental model and study participant details

Female and male *Phlebotomus perniciosus* and *Lutzomyia longipalpis* sand flies were maintained under controlled conditions of 24–28°C, 70–80% relative humidity, and a 12-hour light/dark cycle and used for saliva collection or biting experiments. C57BL/6 and *Ifnar1*^*-*^*/*^*-*^ mice (≥4 weeks old, sex- and age-matched) were used for *in vivo* infections and maintained under specific-pathogen-free conditions. All *in vivo* procedures were undertaken following local ethical (AWERB) and Home Office (HO) approval (Personal License I83228479, Project Licences PP0258562). The effect of mouse sex on infection outcomes was not specifically analysed. Baby hamster kidney-21 (BHK-21) and Vero cells were employed for propagating virus stock and plaque assays. BHK-21 and Vero cells were cultured at 37 °C with 5% CO2 in Dulbecco's Modified Eagle Medium (DMEM) supplemented with 5% fetal calf serum (FCS), 1% tryptose phosphate broth (TPB), 100 units/mL penicillin, 0.1 mg/mL streptomycin and 1% Glutamax. Cell lines were originally derived from ATCC and were not specifically authenticated for this study. Both cell lines were tested for mycoplasma contamination. Primary mouse macrophages and dendritic cells were derived from bone marrow progenitors by culturing with M-CSF and Flt3L, respectively, whereas skin fibroblasts were isolated by enzymatic digestion and MACS-based enrichment.

### Method details

#### Sandfly colonies

Two sand fly species were used: *Phlebotomus perniciosus* (Murcia, Spain) and *Lutzomyia longipalpis* (Jacobina, Bahia state, Brazil). They were maintained at Charles University in Prague and London School of Hygiene and Tropical Medicine, United Kingdom, respectively. Standard methods for sand fly rearing were described previously by Lawyer et al. (2017).^59^ *Lutzomyia longipalpis* females were transferred to School of Medicine, University of Leeds, allowed to rest for one day, followed by a 24-hour starvation period before being used in biting experiments.

#### Virus strains

Virus stocks of Semliki Forest Virus (SFV4) were generated from plasmids containing the genomic sequence, kindly provided by Prof. Andres Merits, University of Tartu. Previously, plasmids had been electroporated into Baby Hamster Kidney (BHK)-21 cells to generate infectious virus. Wild-type Toscana virus (strain 1812) from Italy, a strain known to infect mice, originally isolated from a patient in Italy, was kindly supplied Prof. Maria Grazia Cusi, University of Siena. The genetically modified TOSV (strain 1500590), lineage A, obtained from an infected patient, which is an NSs-deletant rTOSV expressing mCherry, a reporter gene (ΔNSs:mCherry) (Alexander et al., 2020).^29^ SFV4 was diluted in phosphate-buffered saline with bovine serum albumin (PBSA) to 1 x 10ˆ4 plaque-forming units (PFU)/μl for injection. Wild-type TOSV and rTOSV were diluted in phosphate-buffered saline with bovine serum albumin (PBSA) to 1 x 10ˆ5 plaque-forming units (PFU)/μl for injection.

#### Mouse strains

Wild type C57BL/6 mice were bred in the SBS facility at the University of Leeds. *Ifnar1-/-* mice were purchased from the Jackson Laboratory and bred in-house at the SBS at the University of Leeds. Mice were maintained at the SBS under specific pathogen free conditions. All mice were aged at least 4 weeks old and above at time of use, and were age and sex matched for experiments. All *in vivo* procedures were undertaken following local ethical (AWERB) and Home Office (HO) approval (Personal License I83228479, Project Licences PP0258562).

#### Cell culture

Cells were kept at -196°C for long-term storage. Baby hamster kidney-21 (BHK-21) and Vero cells were used to grow up virus stock and determining viral titers via plaque assays. BHK-21 and Vero cells were cultured at 37°C with 5% CO2 in Dulbecco's Modified Eagle Medium (DMEM) supplemented with 5% fetal calf serum (FCS), 1% tryptose phosphate broth (TPB), 100 units/mL penicillin, 0.1 mg/mL streptomycin and 1% Glutamax. Mouse leukocytes were differentiated from bone marrow precursors by culturing with specific cytokines: macrophages with M-CSF (10 ng/ml) for 6 days and dendritic cells (DCs) with Flt3L (200 ng/ml) for 10 days. Skin fibroblasts were derived from adult mouse skin by enzymatic digestion using collagenase D (1 mg/ml), dispase II (0.5 mg/ml), and DNase (0.1 mg/ml) in Hanks’ Balanced Salt Solution (HBSS). The isolated cells were cultured in flasks pre-coated with 0.2% gelatin and maintained in complete DMEM (10% FCS), allowing adherent fibroblasts to proliferate and become the dominant surviving cell population.

**Magnetic-activated Cell Sorting (MACS)** for Murine Macrophage and Fibroblast Isolation from Skin. Single-cell suspensions were prepared from skin samples using enzymatic digestion. Cells were kept cold with pre-cooled solutions to prevent antibody capping and non-specific binding. Cell concentration was determined using a hemacytometer. Suspensions were centrifuged at 300xg for 10 min, and the supernatant was discarded. Macrophage isolation: cells were resuspended in 90μl of MACS buffer (PBS [-Ca/Mg], 1% FBS, 2 mM EDTA) per 10^7^ cells. Anti-F4/80 MicroBeads UltraPure (10μl per 10^7^ cells; Miltenyi Biotec, Germany) were added, mixed, and incubated at 4°C for 15 min. After washing, the cell suspension was applied to an MS column placed in a MACS Separator. Unlabelled cells were collected as flow-through, while bound macrophages (F4/80^+^) were eluted after removing the column from the separator. Fibroblast isolation: fibroblasts were isolated using the Tumour-Associated Fibroblast Isolation Kit (Miltenyi Biotec, Germany) from skin inoculation site. Non-fibroblasts were first depleted using a cocktail of antibodies against non-tumour fibroblasts, followed by magnetic separation with LD columns. The flow-through fraction, containing enriched fibroblasts, was collected. For positive selection, fibroblasts were labelled with CD90.2 MicroBeads (20μl per 10^7^ cells) and incubated at 4°C for 15 min. After washing, cells were applied to an MS column, and unlabelled cells were removed as flow-through. The column was then flushed to elute CD90.2^+^ fibroblasts.

#### Adult sandfly holding

Rearing and Handling. *Lutzomyia longipalpis* sandfly species (Jacobina, Bahia state, Brazil) were kindly provided by Dr. Matthew Rogers (London School of Hygiene and Tropical Medicine, United Kingdom). The sand flies were housed in large fabric-net adult holding cages (30 × 30 × 30 cm) suspended on a metal frame. They were maintained under controlled conditions of 24–28°C, 70–80% relative humidity, and a 12-hour light/dark cycle. To sustain energy and longevity, cotton balls saturated with 30–50% sucrose solution were placed on the cage screen tops as a sugar source. Upon arrival at our facility, the sand flies were allowed to rest for one day, followed by a 24-hour starvation period before being used in biting experiments.

#### Obtaining sandfly salivary gland extract

*Phlebotomus perniciosus* (Murcia, Spain) salivary gland extract (SGE) was kindly provided by Petr Volf (Charles University, Czech Republic) in aliquots of 100 glands/100μl or 10 glands/10μl of 0.9% NaCl. *Lutzomyia longipalpis* SGE were kindly provided by Dr. Matthew Rogers (London School of Hygiene and Tropical Medicine, United Kingdom). Four-day-old, non-blood-fed female sand flies were immobilized on ice; Under a dissecting microscope, salivary glands were extracted by carefully removing the head and isolating the glands in phosphate-buffered saline (PBS). The glands were then disrupted by sonication for 10 seconds or two rounds of freeze-thaw to disrupt the glands, followed by centrifugation at 10,000xg for 2 min, and the supernatant was collected. In this study, we use the term ‘SGE’ to describe salivary gland extract obtained by dissection and disruption of glands, ‘saliva’ to refer to the material naturally deposited during sand fly feeding, and ‘bite’ to indicate the live feeding condition.

#### Antibiotic treatment

To generate microbiota-free *Lutzomyia longipalpis* sand flies, we used a similar antibiotic cocktail as previously described (Kelly et al., 2017).^27^ A mixture of Penicillin (500 U/ml), Streptomycin (500 μg/ml), and Gentamicin sulfate (100 μg/ml) was incorporated into a 25% sucrose solution and provided via soaked cotton pads, which were replaced daily. Antibiotic treatment continued until the biting experiment was performed.

#### In vivo mouse infections

Mice were anesthetised using isoflurane (Henry Schein®, United Kingdom) administered via inhalation. SFV4 (10,000 PFU in 1μl), TOSV (strain 1812, 100,000 PFU in 1μl), TOSV (ΔNSs:mCherry, 100,000 PFU in 1μl) in PBSA was injected into the dorsal aspect of left foot skin, with or without the equal volumes of sandfly salivary gland extract. Injections were carried out using custom-made point 4 style 33-gauge microneedles (Hamilton®, Switzerland) and a 5μl volume glass 75 RN Hamilton syringe (Hamilton®, Switzerland). Immediately following injections, mice were placed in their cages and monitored carefully until they had regained consciousness. Occasionally injections ruptured blood vessel, resulting in minor bleeds; samples derived from these mice were removed from the study.

For experiments with biting flies, mice were anaesthetised with 0.1ml/10g of Sedator®/Ketavet via intraperitoneal injection. The mice were placed in a specially prepared box that would protect their entire bodies and allow them to breathe easily. Then, they were placed in the cage with sand flies in a way that only the dorsal side of left or right foot skin of their feet remained exposed. Toes were covered with tape to prevent sand fly biting. Two sand flies were allowed to bite each foot. Sand flies were left to feed until fully engorged. TOSV (strain 1812, 100,000 PFU in 1μl), TOSV (ΔNSs:mCherry, 100,000 PFU in 1μl) were then injected directly at the bite site using Hamilton® needles, as previously described for mosquito bites (Pingen et al., 2016). Mice were then kept warm and monitored regularly until recovery, or for some experiments injected with 0.1ml/10g of Revertor® reversal agent.

#### Gene expression analysis - RNA extraction - RNA purification and quantification

Mice were euthanized and tissues collected, and blood samples were drawn from the ventricles. Tissue samples were preserved in RNAlater at 4°C for at least 16 hours to prevent RNA degradation before being processed or stored at −80°C. Blood samples were centrifuged to collect serum, which was stored at −80°C until further analysis. RNA extractions were undertaken using Invitrogen™ PureLink™ RNA Mini and Micro Kits for tissue and cell samples, respectively, as per manufacturer’s protocol. Tissue samples in RNAlater were homogenized in TRIzol® and shaken with stainless steel beads using a TissueLyser at 50 Hz for 10 min, followed by phase separation with chloroform. RNA from the aqueous phase was purified using Purelink columns with DNase treatment to remove genomic DNA contamination. For cell samples, lysis buffer with β-mercaptoethanol was used, and lysates were processed through QIAshredder columns. On-column DNase treatment was performed. RNA was converted to cDNA using the High-Capacity RNA-to-cDNA Kit (Applied Biosystems™). Reactions were prepared using up to 2 μg of total RNA as per manufacturer’s instructions. The resulting cDNA was diluted 1:5 with nuclease-free water and stored at -20°C for further analysis.

Quantitiative (q) PCR was undertaken using PerfeCTa SYBR® Green FastMix (Quantabio). Each biological replicate was run in at least 3 technical triplicates. A standard curve was generated using a 10-fold serial dilution of a PCR-generated standard, as described (Pingen et al., 2016).^16^ Reactions were performed on an Applied Biosystems QuantStudio™ 7 Flex system with the following cycling conditions: 95°C for 3 minutes, followed by 25–40 cycles of 95°C for 3 seconds and 60°C for 30 seconds, ending with a melt curve analysis to verify primer specificity. The cycle threshold (Ct) values were automatically determined by the QuantStudio software, and relative gene expression was normalized to the 18S housekeeping gene. Data analysis of technical replicates were conducted in Microsoft Excel, calculating median values and normalizing to reference gene. Outlier samples that exhibited >5 fold difference in 18S quantity were removed from the analyses. Primers were designed using Primer3 and shown in Table S1.

#### Flow cytometry

Skin tissue samples were enzymatically digested in HBSS with collagenase D (1 mg/ml), dispase II (0.5 mg/ml), and DNase (0.1 mg/ml) for 50 minutes at 37°C. The enzymatic reaction was halted with serum, followed by cell washing, FcR blocking (Miltenyi Biotec), and staining with antibodies and a viability dye. All antibodies list below. Anti-Rat/Hamster Ig κ/Negative Control Compensation Beads (BD™ CompBeads) were stained and used to optimize fluorescence compensation settings for multicolour flow cytometric analysis. After fixing cells with 4% PFA, cells were run on a CytoFLEX (Beckman Coulter Life Sciences). Data were analysed relying on the principle of gating following data compensation. Gates and regions were defined around cell populations with shared characteristics, typically including forward scatter (FCS), side scatter (SSC), and marker expression (e.g., L/D dye-ve, CD45+ve), to examine and quantify these specific populations. mCherry expression was informed through use of fluorescence minus one (FMO) controls.

#### Serum neutralising assay

To assess neutralizing antibodies in mice serum, serial dilutions were incubated with 1000 PFU/ml TOSV at 37°C for 1 hour. The mixtures were added to BHK-21 cells in 96 well plates and incubated for another hour before adding DMEM. Cytopathic effects were monitored for 1–3 days. Cells were fixed with 10% PFA, stained with 1% crystal violet. ImageJ software (NIH) was used to measure the integrated density (IntDen) for each well regarding the serum dilution folds. The IntDen is the sum of pixel values in the selected area, corresponding to the staining intensity. In this context, a lower integrated density would indicate a higher number of plaques (more viral activity) and vice versa.

#### Plaque assay

Plaque assays were performed to quantify titre of infectious virus in viral stocks and for the quantification of viremia following virus infection of mice. BHK-21 cells at 80% confluency in 12 well plates were infected with serially diluted virus samples for 1 hour. After infection, a 1:1 overlay of 2X MEM and 1.2% Avicel was added. Cells were incubated for 2–3 days before fixation with 10% PFA and staining with 1% toluidine blue or 1% crystal violet. Plaques were counted, and PFU was calculated per ml using the following equation: PFU/ml = average number of plaques (in duplicate)÷(Dilution Factor x Inoculation Volume).

#### Decalcification of mice bone and histological staining

Mice foot with ankle joints were fixed in 4% PFA for 48h, decalcified in 14% EDTA at 4°C for 10 days with solution changes every 2 days, rinsed in distilled H_2_O, and stored in 70% ethanol before paraffin embedding. Six micrometre–thick longitudinal sections of whole foot with ankle joints were stained with haematoxylin and eosin (H&E). Images were acquired using a 20x magnification objective Zeiss Axioscan Z1 and analysed using QuPath software (v0.5.1). Images were enhanced, e.g. with contrast and brightness, to aid image clarity.

#### Endotoxin assay

Endotoxin levels in sandfly salivary gland extract (SGE) were measured using the ToxinSensor™ Chromogenic LAL Endotoxin Assay Kit (GenScript). SGE and endotoxin standards (0.1–1 EU/mL) were incubated with LAL reagent at 37°C, followed by chromogenic substrate addition. Absorbance at 545nm was measured using a Cytation 5 reader, and endotoxin concentrations were determined from a standard curve.

#### RNA-seq

Quality of extracted RNA was assessed using the Agilent Bioanalyzer RNA pico chips, and high quality RNA (RIN > 8.5) was taken forward into RNA-Seq library preparation. Firstly, oligo(dT)-primed, full-length cDNA was synthesised and amplified from total RNA input using the SMART-Seq mRNA kits (Takara Bio), as per the manufacturer's guidelines. cDNA was quantified using the Agilent Bioanalyzer HS DNA chips, and ∼5 ng cDNA was taken into library preparation using the NEBNext Ultra II FS library preparation kits for Illumina (New England Biolabs). Resultant libraries were pooled at equimolar ratios and sent for paired end 150 bp sequencing on an Illumina NovaSeq X by Genewiz from Azenta life Sciences. Raw RNA-seq reads were assessed for quality using FastQC (version 0.12.1) and MultiQC (version 1.25.1), and for potential contaminating sequences using Kraken2 (version 2.1.3). Residual adapters, barcodes, and low quality bases were trimmed from the reads using TrimGalore (version 0.6.10). Reads were mapped to the GRCm39 (GenCode version M36) version of the *Mus musculus* genome using STAR aligner (version 5.1.0) and then quantified using the Salmon pseudo-aligner (version 1.10.3). Differential expression analysis was carried out using DESeq2 (version 1.40.2) in R (version 4.3.1), filtering out genes with a read count of less than 10, and using a fold change threshold of > +/- 1.5x and an adjusted p value (FDR) threshold of < 0.05. Gene Ontology enrichment analysis was performed using the online g:Profiler (version e112_eg59_p19_25aa4782) tool, searching the Ensembl mouse gene IDs for significantly up- and down-regulated genes against the g:Profiler *Mus musculus* database. *All RNASeq data has been deposited in Genban*k, accession number GSE297255.

### Quantification and statistical analysis

qPCR and flow cytometry data were analysed utilizing GraphPad Prism software (Version 10, San Diego, CA, USA). Ordinary-one-way ANOVA was performed for comparisons between more than two groups of normally distributed data, whereas unpaired, two-tail Student’s t test was performed for comparisons between two groups. Due to the occasional non-Gaussian distribution of the virus titres, non-parametric Kruskal-Wallis test with Dunn’s multiple comparison test was used for comparisons between more than two groups, whereas non-parametric Mann-Whitney was performed for comparisons between two groups. In all cases n = number of mice used. The definition of center, and dispersion and precision measures are given in figure legends. Here, for data that is normally distributed, mean average is shown with SD or SEM. For data that does not show normal distribution, median average +/- interquartile range is shown. All plots have statistical significance indicated with ∗p < 0.05, ∗∗p < 0.01, ∗∗∗p < 0.001, ∗∗∗∗p < 0.0001, ns = not significant. RNA-seq data were analysed as described in the method details section.

## References

[1] Ayhan N., Eldin C., Charrel R. (2025). Toscana virus: A comprehensive review of 1381 cases showing an emerging threat in the Mediterranean regions. J. Infect..

[2] Gori Savellini G., Gandolfo C., Cusi M.G. (2020). Epidemiology of Toscana virus in South Tuscany over the years 2011-2019. J. Clin. Virol..

[3] Jancarova M., Polanska N., Volf P., Dvorak V. (2023). The role of sand flies as vectors of viruses other than phleboviruses. J. Gen. Virol..

[4] Maroli M., Feliciangeli M.D., Bichaud L., Charrel R.N., Gradoni L. (2013). Phlebotomine sandflies and the spreading of leishmaniases and other diseases of public health concern. Med. Vet. Entomol..

[5] Keskek Turk Y., Ergunay K., Kohl A., Hughes J., McKimmie C.S. (2024). Toscana virus – an emerging Mediterranean arbovirus transmitted by sand flies. J. Gen. Virol..

[6] Kuhn J.H., Abe J., Adkins S., Alkhovsky S.V., Avšič-Županc T., Ayllón M.A., Bahl J., Balkema-Buschmann A., Ballinger M.J., Kumar Baranwal V. (2023). Annual (2023) taxonomic update of RNA-directed RNA polymerase-encoding negative-sense RNA viruses (realm Riboviria: kingdom Orthornavirae: phylum Negarnaviricota). J. Gen. Virol..

[7] Charrel R.N., Bichaud L., de Lamballerie X. (2012). Emergence of Toscana virus in the mediterranean area. World J. Virol..

[8] Charrel R.N., Gallian P., Navarro-Marí J.-M., Nicoletti L., Papa A., Sánchez-Seco M.P., Tenorio A., de Lamballerie X. (2005). Emergence of Toscana Virus in Europe. Emerg. Infect. Dis..

[9] Maia C. (2024). Sand fly-borne diseases in Europe: epidemiological overview and potential triggers for their emergence and re-emergence. J. Comp. Pathol..

[10] Dersch R., Sophocleous A., Cadar D., Emmerich P., Schmidt-Chanasit J., Rauer S. (2021). Toscana virus encephalitis in Southwest Germany: a retrospective study. BMC Neurol..

[11] Pawar N., Seth A.K. (2025). Chandipura Virus in India: A Comprehensive Epidemiological Review. J. Vector Borne Dis..

[12] Conway M.J., Colpitts T.M., Fikrig E. (2014). Role of the Vector in Arbovirus Transmission. Annu. Rev. Virol..

[13] Pingen M., Schmid M.A., Harris E., McKimmie C.S. (2017). Mosquito Biting Modulates Skin Response to Virus Infection. Trends Parasitol..

[14] Edwards J.F., Higgs S., Beaty B.J. (1998). Mosquito Feeding-Induced Enhancement of Cache Valley Virus (Bunyaviridae) Infection in Mice. J. Med. Entomol..

[15] Le Coupanec A., Babin D., Fiette L., Jouvion G., Ave P., Misse D., Bouloy M., Choumet V. (2013). Aedes Mosquito Saliva Modulates Rift Valley Fever Virus Pathogenicity. PLoS Negl. Trop. Dis..

[16] Pingen M., Bryden S.R., Pondeville E., Schnettler E., Kohl A., Merits A., Fazakerley J.K., Graham G.J., McKimmie C.S. (2016). Host Inflammatory Response to Mosquito Bites Enhances the Severity of Arbovirus Infection. Immunity.

[17] Styer L.M., Lim P.-Y., Louie K.L., Albright R.G., Kramer L.D., Bernard K.A. (2011). Mosquito Saliva Causes Enhancement of West Nile Virus Infection in Mice. J. Virol..

[18] Lefteri D.A., Bryden S.R., Pingen M., Terry S., McCafferty A., Beswick E.F., Georgiev G., Van der Laan M., Mastrullo V., Campagnolo P. (2022). Mosquito saliva enhances virus infection through sialokinin-dependent vascular leakage. Proc. Natl. Acad. Sci. USA.

[19] Agarwal A., Joshi G., Nagar D.P., Sharma A.K., Sukumaran D., Pant S.C., Parida M.M., Dash P.K. (2016). Mosquito saliva induced cutaneous events augment Chikungunya virus replication and disease progression. Infect. Genet. Evol..

[20] Belkaid Y., Kamhawi S., Modi G., Valenzuela J., Noben-Trauth N., Rowton E., Ribeiro J., Sacks D.L. (1998). Development of a Natural Model of Cutaneous Leishmaniasis: Powerful Effects of Vector Saliva and Saliva Preexposure on the Long-Term Outcome of Leishmania major Infection in the Mouse Ear Dermis. J. Exp. Med..

[21] Peters N.C., Egen J.G., Secundino N., Debrabant A., Kimblin N., Kamhawi S., Lawyer P., Fay M.P., Germain R.N., Sacks D. (2008). In Vivo Imaging Reveals an Essential Role for Neutrophils in Leishmaniasis Transmitted by Sand Flies. Science.

[22] Titus R.G., Ribeiro J.M. (1988). Salivary Gland Lysates from the Sand Fly Lutzomyia longipalpis Enhance Leishmania Infectivity. Science.

[23] Cusi M.G., Gori Savellini G., Terrosi C., Di Genova G., Valassina M., Valentini M., Bartolommei S., Miracco C. (2005). Development of a mouse model for the study of Toscana virus pathogenesis. Virology.

[24] Lazear H.M., Govero J., Smith A.M., Platt D.J., Fernandez E., Miner J.J., Diamond M.S. (2016). A Mouse Model of Zika Virus Pathogenesis. Cell Host Microbe.

[25] Lestinova T., Rohousova I., Sima M., de Oliveira C.I., Volf P. (2017). Insights into the sand fly saliva: Blood-feeding and immune interactions between sand flies, hosts, and Leishmania. PLoS Negl. Trop. Dis..

[26] Dey R., Joshi A.B., Oliveira F., Pereira L., Guimarães-Costa A.B., Serafim T.D., de Castro W., Coutinho-Abreu I.V., Bhattacharya P., Townsend S. (2018). Gut Microbes Egested during Bites of Infected Sand Flies Augment Severity of Leishmaniasis via Inflammasome-Derived IL-1β. Cell Host Microbe.

[27] Kelly P.H., Bahr S.M., Serafim T.D., Ajami N.J., Petrosino J.F., Meneses C., Kirby J.R., Valenzuela J.G., Kamhawi S., Wilson M.E. (2017). The gut microbiome of the vector lutzomyia longipalpis is essential for survival of leishmania infantum. mBio.

[28] Wang Z., Nie K., Liang Y., Niu J., Yu X., Zhang O., Liu L., Shi X., Wang Y., Feng X. (2024). A mosquito salivary protein-driven influx of myeloid cells facilitates flavivirus transmission. EMBO J..

[29] Alexander A.J.T., Confort M.P., Desloire S., Dunlop J.I., Kuchi S., Sreenu V.B., Mair D., Wilkie G.S., da Silva Filipe A., Brennan B. (2020). Development of a reverse genetics system for Toscana virus (Lineage A). Viruses.

[30] Jiang D., Guo R., Machens H.G., Rinkevich Y. (2023). Diversity of Fibroblasts and Their Roles in Wound Healing. Cold Spring Harb. Perspect. Biol..

[31] Plikus M.V., Wang X., Sinha S., Forte E., Thompson S.M., Herzog E.L., Driskell R.R., Rosenthal N., Biernaskie J., Horsley V. (2021). Fibroblasts: Origins, definitions, and functions in health and disease. Cell.

[32] Bautista-Hernández L.A., Gómez-Olivares J.L., Buentello-Volante B., Bautista-de Lucio V.M. (2017). Fibroblasts: the unknown sentinels eliciting immune responses against microorganisms. Eur. J. Microbiol. Immunol..

[33] Rong L., Liu J., Qi Y., Graham A.M., Parmacek M.S., Li S. (2012). GATA-6 promotes cell survival by up-regulating BMP-2 expression during embryonic stem cell differentiation. Mol. Biol. Cell.

[34] Saito S., Kitabatake M., Ouji-Sageshima N., Ogawa T., Oda A., Nishimura T., Nishioka T., Fushimi S., Hara A., Shichino S. (2023). Angiopoietin-like 4 Is a Critical Regulator of Fibroblasts during Pulmonary Fibrosis Development. Am. J. Respir. Cell Mol. Biol..

[35] Ushakumary M.G., Green J., Riccetti M.R., Na C.-L., Mohanraj D., Guo M., Perl A.-K.T. (2022). Matrix fibroblast function during alveolarization is dependent on GATA6.

[36] Zhang H., Qiu J., Zhao Q., Zhang Y., Zheng H., Dou Z., Yan Y. (2024). Tanshinone IIA alleviates bleomycin-induced pulmonary fibrosis by inhibiting Zbtb16. Pulm. Pharmacol. Ther..

[37] Bielli A., Scioli M.G., D’Amico F., Tarquini C., Agostinelli S., Costanza G., Doldo E., Campione E., Passeri D., Coniglione F., Orlandi A. (2019). Cellular retinoic acid binding protein-II expression and its potential role in skin aging. Aging.

[38] Martinez-Ferrer M., Afshar-Sherif A.R., Uwamariya C., De Crombrugghe B., Davidson J.M., Bhowmick N.A. (2010). Dermal transforming growth factor-β responsiveness mediates wound contraction and epithelial closure. Am. J. Pathol..

[39] Repertinger S.K., Campagnaro E., Fuhrman J., El-Abaseri T., Yuspa S.H., Hansen L.A. (2004). EGFR Enhances Early Healing After Cutaneous Incisional Wounding. J. Invest. Dermatol..

[40] Watterson K.R., Lanning D.A., Diegelmann R.F., Spiegel S. (2007). Regulation of fibroblast functions by lysophospholipid mediators: Potential roles in wound healing. Wound Repair Regen..

[41] Yamada M., Masai H., Bartek J. (2014). Regulation and roles of Cdc7 kinase under replication stress. Cell Cycle.

[42] Nguyen X.X., Muhammad L., Nietert P.J., Feghali-Bostwick C. (2018). IGFBP-5 promotes fibrosis via increasing its own expression and that of other pro-fibrotic mediators. Front. Endocrinol..

[43] Fragkoudis R., Tamberg N., Siu R., Kiiver K., Kohl A., Merits A., Fazakerley J.K. (2009). Neurons and oligodendrocytes in the mouse brain differ in their ability to replicate Semliki Forest virus. J. Neurovirol..

[44] Oliver K.R., Scallan M.F., Dyson H., Fazakerley J.K. (1997). Susceptibility to a neurotropic virus and its changing distribution in the developing brain is a function of CNS maturity. J. Neurovirol..

[45] Tang H., Hammack C., Ogden S.C., Wen Z., Qian X., Li Y., Yao B., Shin J., Zhang F., Lee E.M. (2016). Zika virus infects human cortical neural progenitors and attenuates their growth. Cell Stem Cell.

[46] Driskell R.R., Lichtenberger B.M., Hoste E., Kretzschmar K., Simons B.D., Charalambous M., Ferron S.R., Herault Y., Pavlovic G., Ferguson-Smith A.C., Watt F.M. (2013). Distinct fibroblast lineages determine dermal architecture in skin development and repair. Nature.

[47] Jiang D., Rinkevich Y. (2018). Defining skin fibroblastic cell types beyond CD90. Front. Cell Dev. Biol..

[48] Nazari B., Rice L.M., Stifano G., Barron A.M.S., Wang Y.M., Korndorf T., Lee J., Bhawan J., Lafyatis R., Browning J.L. (2016). Altered Dermal Fibroblasts in Systemic Sclerosis Display Podoplanin and CD90. Am. J. Pathol..

[49] Fotakis E.A., Di Maggio E., Del Manso M., Mateo-Urdiales A., Petrone D., Fabiani M., Perego G., Bella A., Bongiorno G., Bernardini I. (2025). Human neuroinvasive Toscana virus infections in Italy from 2016 to 2023: Increased incidence in 2022 and 2023. Euro Surveill..

[50] Jaijakul S., Arias C.A., Hossain M., Arduino R.C., Wootton S.H., Hasbun R. (2012). Toscana meningoencephalitis: A comparison to other viral central nervous system infections. J. Clin. Virol..

[51] Vilibic-Cavlek T., Zidovec-Lepej S., Ledina D., Knezevic S., Savic V., Tabain I., Ivic I., Slavuljica I., Bogdanic M., Grgic I. (2020). Clinical, virological, and immunological findings in patients with toscana neuroinvasive disease in Croatia: Report of three cases. Trop. Med. Infect. Dis..

[52] Cusi M.G., Gandolfo C., Terrosi C., Gori Savellini G., Belmonte G., Miracco C. (2016). Toscana virus infects dendritic and endothelial cells opening the way for the central nervous system. J. Neurovirol..

[53] Quicke K.M., Bowen J.R., Johnson E.L., McDonald C.E., Ma H., O’Neal J.T., Rajakumar A., Wrammert J., Rimawi B.H., Pulendran B. (2016). Zika Virus Infects Human Placental Macrophages. Cell Host Microbe.

[54] Schmid M.A., Diamond M.S., Harris E. (2014). Dendritic cells in dengue virus infection: Targets of virus replication and mediators of immunity. Front. Immunol..

[55] Samuel M.A., Diamond M.S. (2006). Pathogenesis of West Nile Virus Infection: a Balance between Virulence, Innate and Adaptive Immunity, and Viral Evasion. J. Virol..

[56] Kamhawi S., Belkaid Y., Modi G., Rowton E., Sacks D. (2000). Protection Against Cutaneous Leishmaniasis Resulting from Bites of Uninfected Sand Flies. Science.

[57] Schneider B.S., Soong L., Girard Y.A., Campbell G., Mason P., Higgs S. (2006). Potentiation of West Nile Encephalitis by Mosquito Feeding. Viral Immunol..

[58] Visser I., Vaes V., van Run P., Marshall E.M., Vermaat L., Linthout C., Dekkers D.H.W., Demmers J.A.A., Koopmans M.P.G., Koenraadt C.J.M. (2025). Effect of mosquito saliva from distinct species on human dermal endothelial cell function in vitro and West Nile virus pathogenesis in vivo. Emerg. Microbes Infect..

[59] Lawyer P., Killick-Kendrick M., Rowland T., Rowton E., Volf P. (2017). Laboratory colonization and mass rearing of phlebotomine sand flies (Diptera, Psychodidae). Parasite.

