# Supplementary material for: Sand fly saliva reprograms skin fibroblasts to enhance arbovirus infection
Source: iScience. 2025 Oct 25;28(11):113854. doi: 10.1016/j.isci.2025.113854 (PMC12682282; doi:10.1016/j.isci.2025.113854)
Supplement: Document S1. Figures S1–S8 and Table S1 [file mmc1.pdf]

iScience, Volume *28*

Supplemental information

Sand fly saliva reprograms

skin fibroblasts to enhance arbovirus infection

Yonca Keskek Turk, Ailish McCafferty-Brown, Liam Barningham, Magdalena Jancarova, Petr Volf, Matthew E. Rogers, Akira J.T. Alexander, Çağdaş Kaya, Sandy MacDonald, Maria Grazia Cusi, Alain Kohl, Kave Shams, and Clive S. McKimmie

Supplementary figures

Figure S1

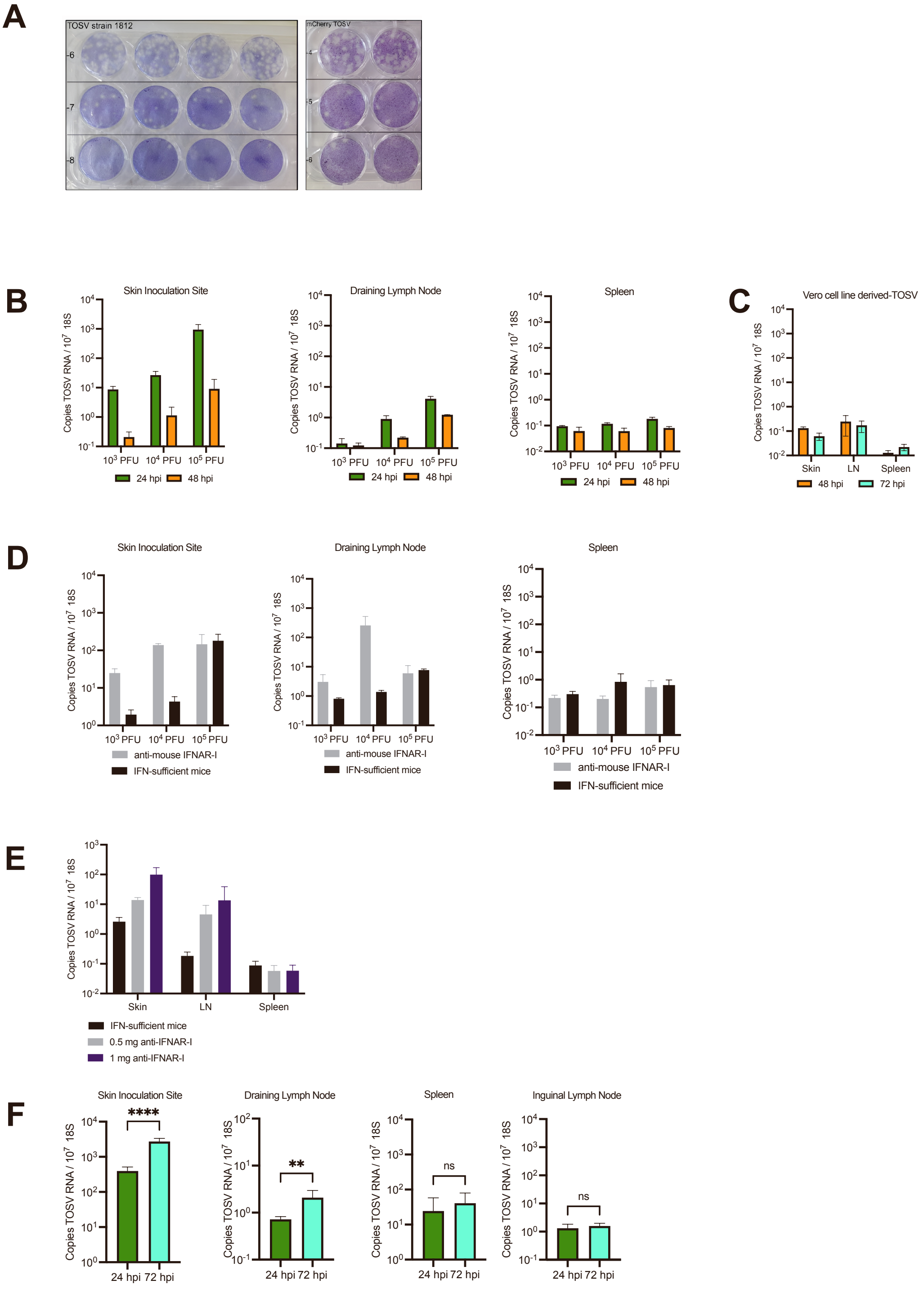

Figure S1. Optimisation of TOSV mouse model.

(A) S1A: Example of TOSV (strain 1812V) and TOSV-mCherry plaque assay result.

(B,C) C57BL/6 mice were infected with either (B) 1000, 10,000, or 100,000 PFU of TOSV (BHK cell-derived) or (C) 100,000 PFU TOSV (Vero cell-derived). Tissues were taken from infected mouse at either 24hpi, 48hpi or 72 hpi. TOSV RNA (Ns gene) copy number was determined by qPCR. Blood was also collected to assess quantity of infectious units by plaque assay (no detectable plaques).

(D) C57BL/6 mice were either left resting or administered subcutaneously with 499.5 µg of InVivoMAb anti-mouse IFNAR-blocking antibody 24 hours prior to infection and then infected with either 1000, 10,000, 100,000 PFU of TOSV (BHK cell-derived). Tissues were taken from infected mouse at 24hpi and TOSV RNA (Ns gene) copy number determined by qPCR. Blood was also collected to assess quantity of infectious units by plaque assay (no detectable plaque).

(E) C57BL/6 mice treated with IFNAR-1 a day before infection were infected with 10,000 PFU of TOSV subcutaneously. Tissues were taken from infected mice at 24hpi. TOSV RNA (Ns gene) copy number was determined by qPCR. Blood was also collected to assess quantity of infectious units by plaque assay (no detectable plaque).

(F) *Ifnar1*-/- mice (n=5) were infected with 100,000 PFU of TOSV (BHK cell-derived). Tissues were taken at 24hpi and 72hpi and TOSV RNA (Ns gene) copy number determined by qPCR. Blood was also collected to assess quantity of infectious units by plaque assay (no detectable plaque). Draining lymph nodes were popliteal, and non-draining lymph nodes were inguinal.

All columns represent the mean value ± SD. ns=not significant, significant *$P < 0.05$, ****$P < 0.0001$ using unpaired Student's two-tailed t test.

Figure S2

A

PANEL 1

Antibody	Clone	Fluorophore	Marker for
CD45	30-F11	FITC	common leukocyte antigen
CD11b	M1/70	APC	monocytes, macrophages
MERTK (Mer)	2B10C42	APC-Cy7	macrophages
Ly-6G	1A8	Brilliant Violet 421	neutrophils
TOSV	n/a	mCherry	infected TOSV cells
Live/Dead dye	n/a	Ultraviolet	cell viability

PANEL 2

Antibody	Clone	Fluorophore	Marker for
CD45	30-F11	FITC	common leukocyte antigen
Ly-6C	HK1.4	PE	monocytes
MHC-II	M5/114.15.2	APC	antigen-presenting cells (APCs)
CD11c	N418	PE-Cy7	dendritic cells (DCs)
TOSV	n/a	mCherry	infected TOSV cells
Live/Dead dye	n/a	Ultraviolet	cell viability

B

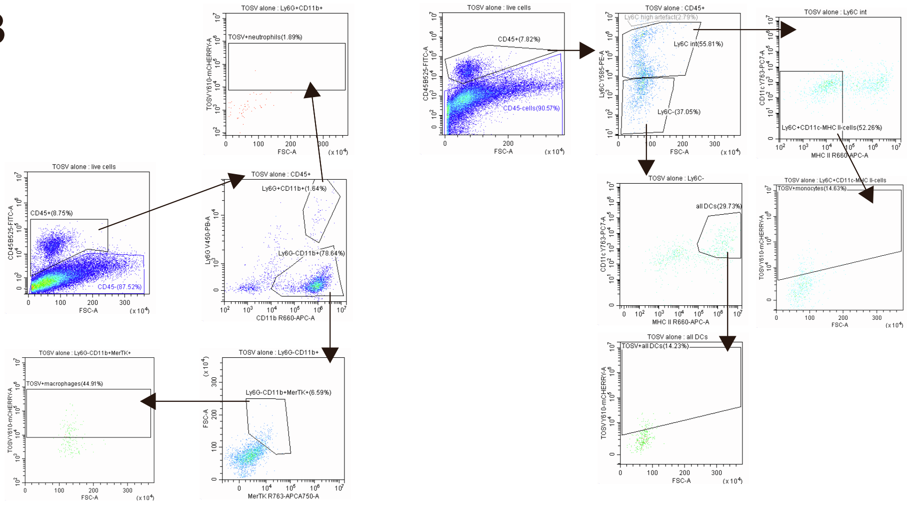

C

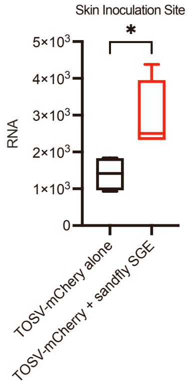

Figure S2. Flow cytometry gating to define mCherry-TOSV infected cells. (A) Antibodies used to stain cells. (B) Gating strategy to define $CD45^{+}CD11b^{hi}$ $Ly6G^{hi}$ neutrophils, $CD45^{+}CD11b^{+}MerTK^{+}FSC^{hi}$ macrophages, $CD45^{+}Ly6C^{+}MHC\text{-}II^{-ve}CD11c^{-ve}$ monocytes, and $CD45^{+}Ly6C^{-ve}MHC\text{-}II^{hi}CD11c^{+ve}$ dendritic cells (DC). This was representative of n=6. (C) Ifnar1-/- mice were infected with 5000 PFU TOSV-mCherry with or without sandfly SGE (1 pair gland extract). Skin samples were taken at 72hpi and TOSV RNA (N gene) copy number determined by qPCR (n=4 mice). Plots show the median value ± interquartile range. Significant, *P < 0.05 using unpaired Student's two-tailed t test.

Figure S3.

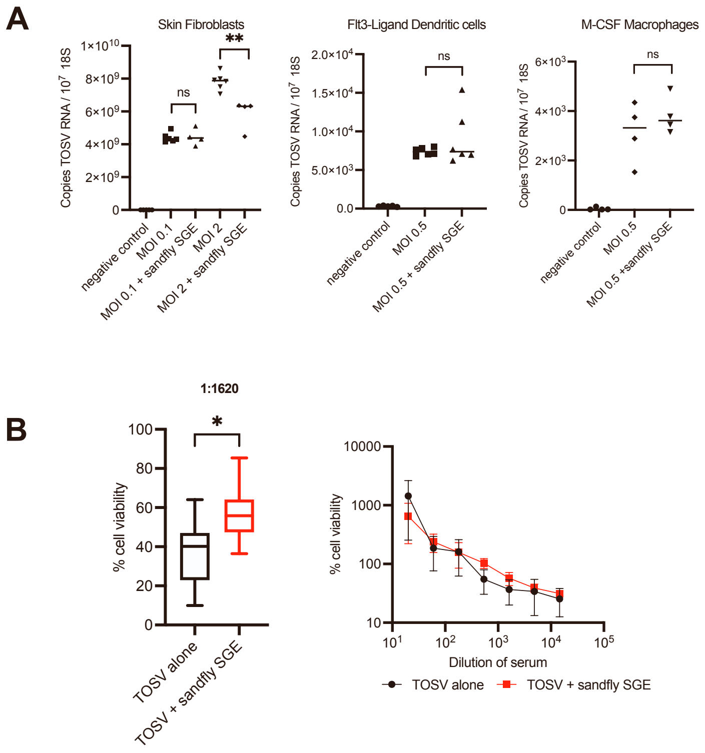

Figure S3. Sandfly SGE does not modulate infection of primary cell cultures by TOSV in vitro.

(A) TOSV (strain 1812V) infection with an MOI of either 0.1 or 2 for cultured primary fibroblast cultures (n=5) and MOI of 0.5 for macrophages (n=4) and dendritic cells (n=6), either on its own or following pre-treatment with one pair of sandfly SGE for 20 minutes before infection. The cells were collected 24hpi and TOSV RNA (Ns gene) copy number was determined by qPCR assay. Data is presented as dot plots with each dot representing a separate biological sample with a line at the population median. ns=not significant, significant **P < 0.01.

(B) 1000 PFU of TOSV was pre-treated with serum collected from *ifnar1-/-* mice infected with either TOSV-alone, or TOSV with sandfly SGE, taken at 3 weeks post-infection. Serum was firstly diluted, ranging from 1:20 to 1:14580. Following one hour of incubation, BHK cells were infected with these mixtures of virus and serum and observed over the 3 few days. After observing cytopathic effects (CPE), the cells were stained with crystal violet to identify which wells were CPE+ve. The plates were then scanned and analysed using ImageJ to calculate the percentage coverage of viable cells, serving as a measure of the serum's protective capacity. All cell viability measurements were normalised against background median value of uninfected cells. Plots show the median value ± interquartile range. Significant, *P < 0.05 using unpaired Student's two-tailed t test. The whisker plot shows percentage of viable cells when treated with serum diluted 1:1620. The line graph illustrates percentage of cell viability in a range of dilutions between 1:20 and 1:14580.

Figure S4

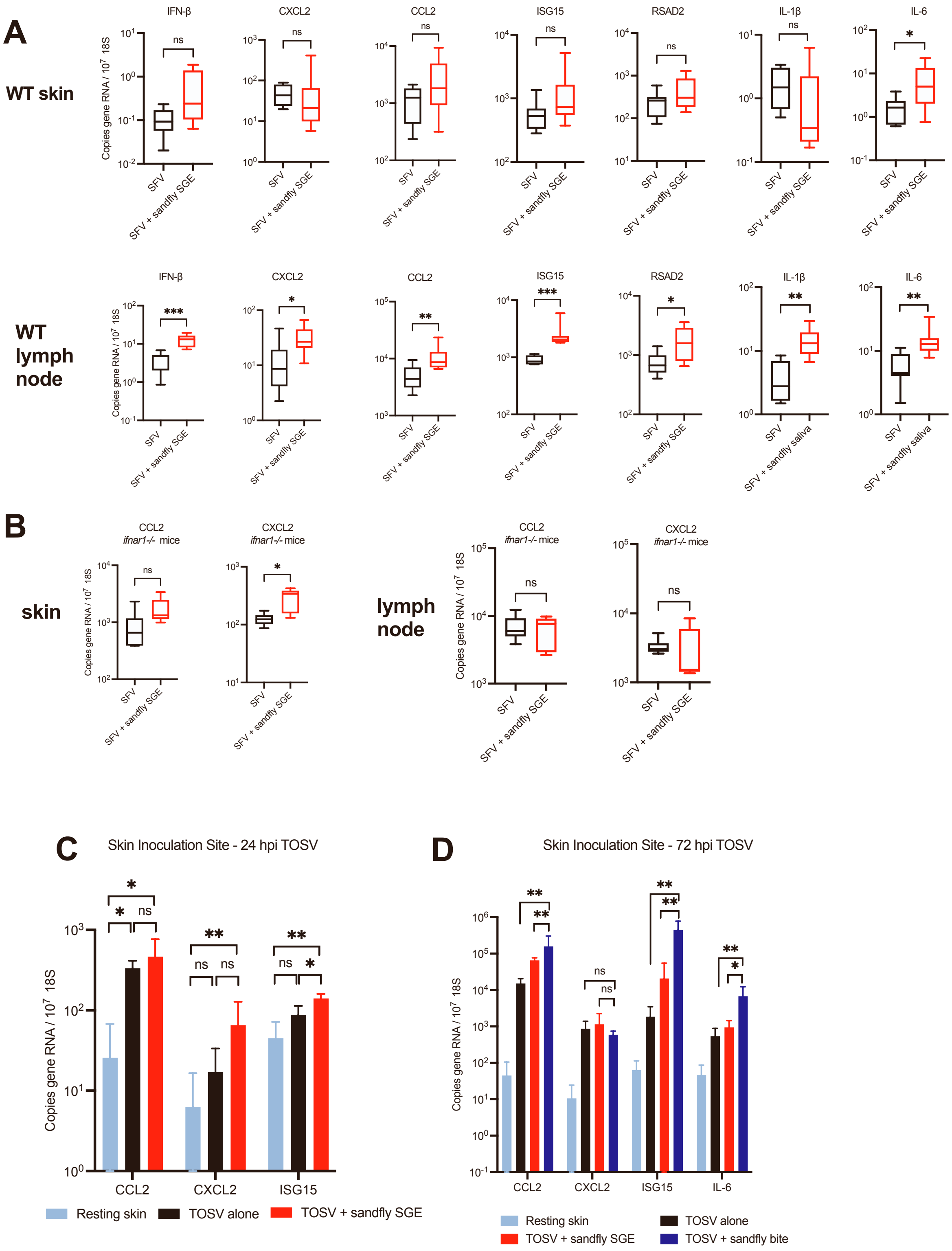

Figure S4 – Sandfly SGE enhances expression of pro-inflammatory cytokine and chemokine response to TOSV infection in mice.

(A) Wild type mouse (n=8) skin was injected with either 10^4 PFU SFV alone or co-injected with 1 salivary gland pair SGE. (B) *Ifnar1*-/- mouse (n=8) skin was injected with either 10^4 PFU SFV alone or co-injected with 1 salivary gland pair SGE. Plots show the median value ± interquartile range. Significant, *P < 0.05, **P<0.01 using Mann Whitney.

(C,D) *Ifnar1*-/- mouse (n=5) skin was injected with either 10^5 PFU TOSV alone or co-injected with 1 salivary gland pair SGE. (D) *Ifnar1*-/- mouse (n=6) skin was either injected with 10^5 PFU TOSV alone, or co-injected with 1 salivary gland pair SGE, or exposed to sand fly biting and injected with 10^5 PFU TOSV. Columns represent mean +/- SD. Significant, *P < 0.05 using Kruskal Wallis test.

(A-C) At 24 hpi and (D) 72 hpi, gene transcripts were quantified by qPCR.

Figure S5

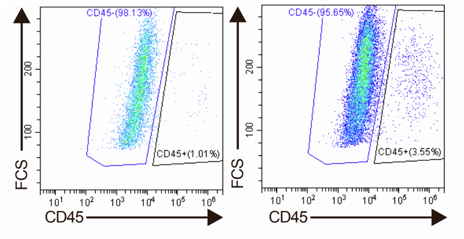

Figure S5 – SGE alone recruits CD45+ leukocytes to skin inoculation site.

Ifnar1-/- mouse skin was injected with either saline control (left) or 1 salivary gland pair of SGE (right), and frequency of CD45+ leukocytes defined by flow cytometry at 72 hours post administration.

Figure S6 – flow cytometry panel used to define fibroblast sub-populations.

Antibody	Clone	Fluorophore	Marker for
CD45	30-F11	FITC	common leukocyte antigen
Vimentin	280618	APC	mesenchymal cells, including fibroblasts
CD31	390	PE	pan-endothelial cells
CD326 (Ep-CAM)	G8.8	APC-Cy7	epithelial cells, including keratinocytes
TOSV	n/a	mCherry	infected TOSV cells
Live/Dead dye	n/a	Ultraviolet	cell viability

FIBROBLAST SUBPOPULATIONS PANEL

Antibody	Clone	Fluorophore	Marker for
CD45	30-F11	FITC	common leukocyte antigen
CD31	390	FITC	endothelial cells
CD326 (Ep-CAM)	G8.8	FITC	epithelial cells
CD90.2 (Thy1.2)	30-H12	Pacific Blue	fibroblasts
Podoplanin	PMab-1	Alexa Fluor 647	activated fibroblasts
CD140a (PDGFR-α)	APA5	PE-Cy7	activated fibroblasts
Ly-6A/E (Sca-1)	W18174A	PE	pluripotent mesenchymal
TOSV	n/a	mCherry	infected TOSV cells
Live/Dead dye	n/a	Ultraviolet	cell viability

Ifnar1-/- mouse skin (n=6) was infected with $5x10^3$ PFU mCherry-TOSV with *P. pernicious* SGE and at 72 hours skin cells stained for flow cytometry. Single live cells were gated to define CD45, EpCAM and CD31 positive cells (lineage positive) and fibroblast containing gate (lineage negative cells). Lineage negative gate was then further analysed to define cells double positive for mCherry-TOSV and either Podoplanin, CD140a, Sca-1 and CD90.2.

Figure S7

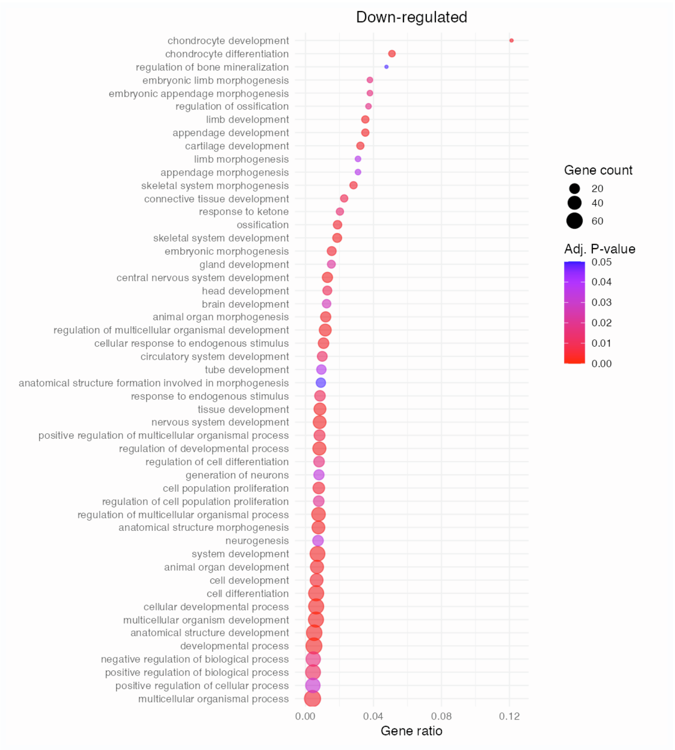

Figure S7. GO frequency for developmentally related descriptors were down regulated by sand fly SGE.

Ifnar1-/- mouse skin was injected with sand fly SGE and at 72 hours skin gene expression was assessed by RNA-seq. Here, identified down-regulated DEG were utilised to generated a bubble plot showing GO descriptors.

Figure S8

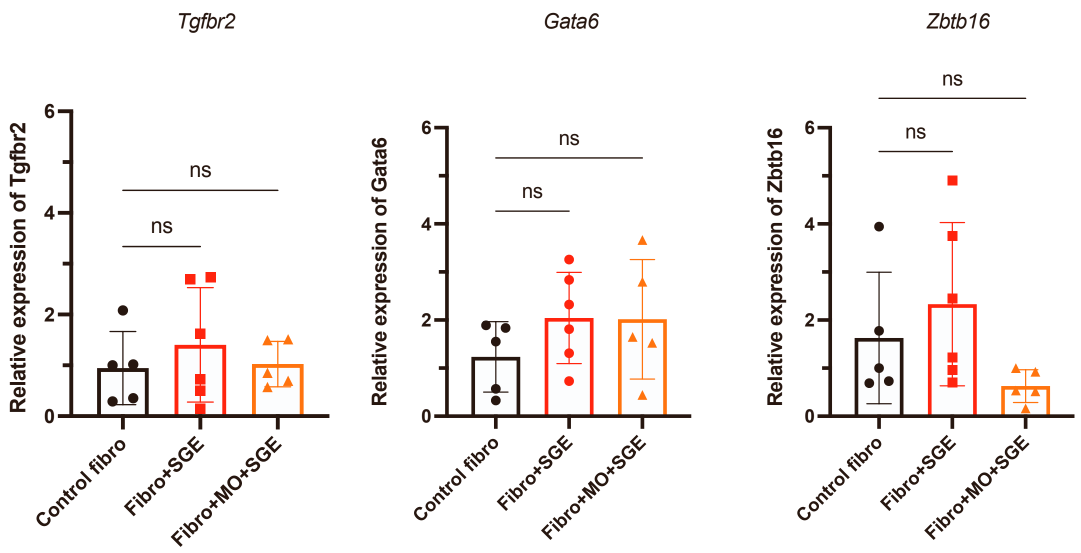

Supplementary figure 8. *In vitro* stimulation of fibroblasts with SGE does not upregulate expression of Tgfbr2, Gata6 or Zbtb16.

To assess whether SGE could have direct effects on fibroblasts, we stimulated mouse skin-derived cultures of fibroblasts with SGE for 72 hours and assess expression of 3 gene identified in our RNA-seq analysis, by qPCR (n=6). To additional define whether macrophages, which are recruited by SGE *in vivo*, could also help induce fibroblast gene expression change, we additionally stimulated fibroblasts cultures with SGE in presence of bone-marrow derived primary macrophages (at 1:5 ratio, n=6). Fibroblasts were derived as per protocol and cultured for 7 days, and then cultured for 24 hours of in low serum conditions prior to SGE stimulation (2% FCS). ns = non-significant according to ordinary one-way ANOVA with Dunnett's multiple comparison test.

Supplementary table 1

List of primers, primer orientation, sequences, product size and NCBI reference.

Gene Name	Orientation	Sequence	Product size (bp)	NCBI Reference
18S	Forward	gactcaacacgggaaacctc	124	NR_003278. 1
	Reserve	taaccagacaaatcgctccac		
18S Standard	Forward	cgtagttccgaccataaacga	443	NR_003278. 1
	Reserve	acatctaagggcatcacagacc		
TOSV Ns	Forward	gaaccagactttacgagccaac	102	X53794.1
	Reserve	gccacctgagagcagacaa		
TOSV Ns Standard	Forward	ggttcaggccacaagaggt	519	X53794.1
	Reserve	agcagtcaatccgtgctttt		
SFV E1	Forward	cgcatcaccttcttttgtg	173	DQ_189086
	Reserve	ccagaccacccgagatttt		
SFV E1 Standard	Forward	aagtgaagacagcaggtaaggtg	446	DQ_189086
	Reserve	tatgagttgccccgagtttc		
CXCL10	Forward	tgccacgatgaaaaagaatg	182	NM_021274
	Reserve	aggggagtgatggagagagg		
CXCL10 Standard	Forward	atccctgcgagcctatcc	524	NM_021274
	Reserve	aaacttagaactgacgagcctga		
IFN-β	Forward	cacagccctctccatcaact	152	NM_010510
	Reserve	gcatcttctccgtcatctcc		
IFN-β Standard	Forward	ggcttccatcatgaacaaca	399	NM_010510
	Reserve	tcccacgtcaatctttcctc		
Rsad2	Forward	tgaagcgtggcggaaagtat	73	NM_021384.4
	Reserve	tccttcccatctcagcctca		
Rsad2 Standard	Forward	ctgtgcgctggaaggttttc	583	NM_021384.4
	Reserve	cactggaccttgctcctctg		
ISG15	Forward	cgcagactgtagacacgctta	80	NM_015783.3
	Reserve	ctcgaagctcagcagaact		
ISG15 Standard	Forward	gtccgtgactaactccatgac	504	NM_015783.3
	Reserve	tcccaaaagtcctccatacc		
CXCL2	Forward	aagtttgccttgaccctgaa	129	NM_009140

	Reserve	tctctttggttcttccgttg		
CXCL2 Standard	Forward	cgcccagacagaagtcatag	484	NM_009140
	Reserve	actcaccctctccccagaaa		
IL-1β	Forward	cgctcagggtcacaagaaac	67	NM_008361.3
	Reserve	gaggcaaggaggaaaacaca		
IL-1β standard	Forward	aaagtatgggctggactgtttc	410	NM_008361.3
	Reserve	atgtgctggtgcttcattca		
TBP	Forward	tgctgttggtgattgttggt	99	NM_013684
	Reserve	aactggcttgtgtgggaaag		
TBP Standard	Forward	gagttgcttgctctgtgctg	274	NM_013684
	Reserve	atactgggaaggcggaatgt		
CCL2	Forward	ctcacctgctgctactcattca	153	NM_011333.3
	Reserve	ccattccttcttggggtca		
CCL2 standard	Forward	caccagcaccagccaact	519	NM_011333.3
	Reserve	gcatcacagtccgagtcaca		
TNF-α	Forward	caccaccatcaaggactcaa	96	NM_013693
	Reserve	gaggcaacctgaccactctc		
TNF-α Standard	Forward	tctgtgaagggaatgggtgt	420	NM_013693
	Reserve	ggctggctctgtgaggaa		
IL-6	Forward	ttccatccagttgccttctt	171	NM_031168
	Reserve	atttccacgatttcccagag		
IL-6 Standard	Forward	tccagaaaccgctatgaagt	370	NM_031168
	Reserve	ctccagaagaccagaggaaa		
